# AWaRe antibiotic prescribing for common acute infections in private primary care in low–middle-income countries: a patient-level analysis using IQVIA prescriber surveys from Pakistan, Egypt and Indonesia

**DOI:** 10.1136/bmjgh-2025-021139

**Published:** 2026-05-07

**Authors:** Nam Nguyen, Sherry Mangla, Peter Stephens, Aislinn Cook, Mike Thorn, Zikria Saleem, Siswanto Agus Wilopo, Elizabeth Tayler, Mike Sharland, Koen B Pouwels

**Affiliations:** 1 Nuffield Department of Primary Care Health Sciences, University of Oxford, Oxford, UK; 2 Antibiotic Policy Group, Institute of Infection and Immunity, School of Health & Medical Sciences, City St George’s University of London, London, England, UK; 3 IQVIA Commercial GmbH and Co OHG, Frankfurt, Germany; 4 IQVIA Ltd, London, England, UK; 5 Department of Pharmacy Practice, College of Pharmacy, Qassim University, Buraydah, Al Qassim, Saudi Arabia; 6 Department of Biostatistics, Epidemiology, and Population Health, Faculty of Medicine, Public Health and Nursing, Universitas Gadjah Mada, Yogyakarta, Jogja, Indonesia; 7 Centre for Reproductive Health, Faculty of Medicine, Public Health and Nursing, Universitas Gadjah Mada, Yogyakarta, Jogja, Indonesia

**Keywords:** Global Health, Health policies and all other topics, Infections, diseases, disorders, injuries, Antimicrobial Stewardship

## Abstract

**Backgrounds:**

There is limited high-quality data on antibiotic prescribing in low and middle-income countries, particularly in the private sector. Here, we use large-scale healthcare surveys to assess antibiotic prescribing levels and the factors influencing prescribing decisions for common infections in primary care and outpatient settings, predominantly within the private sector, in Pakistan, Egypt and Indonesia.

**Methods:**

We analysed surveys completed by prescribers in Pakistan, Egypt and Indonesia, collected in primary care and outpatient settings, predominantly within the private sector, by IQVIA between 2017 and 2021, namely IQVIA’s proprietary Medical Data Index (Medical Index of Pakistan (MIP), Egypt Medical Data Index (EMDI) and Indonesia Medical Data Index (IMDI)). IQVIA market research information reflects estimates of real-world activity and should be treated accordingly. We evaluated antibiotic prescribing categorised by WHO AWaRe and Essential Medicines List (EML) classifications for common infections. We used mixed-effects regression analyses to identify factors influencing prescribing decisions.

**Results:**

Among the 384 975 infection-related health consultation records analysed, antibiotics were prescribed in 82.0% of consultations in Pakistan, 81.2% in Egypt and 69.1% in Indonesia. Watch antibiotics accounted for 70.2% of antibiotic prescriptions in Pakistan, 52.9% in Egypt and 53.6% in Indonesia. Non-WHO EML antibiotics accounted for 26.8% of prescriptions in Pakistan, 39.9% in Egypt and 33.0% in Indonesia. Consultations for patients presenting with lower respiratory tract infections, urinary tract infections, multiple infections or differentiated fever had higher odds of receiving any or a Watch antibiotic. Consultations by respiratory-related specialists in Pakistan and Egypt and by most specialities in Indonesia were more likely to receive Watch antibiotics.

**Conclusions:**

Similar patterns of high levels of total and Watch antibiotic prescribing for common infections—including those that generally do not require any antibiotics—were identified among prescribers in primary care and outpatient settings within the private sector in Pakistan, Egypt and Indonesia.

**WHAT IS ALREADY KNOWN ON THIS TOPIC**

**WHAT THIS STUDY ADDS**

- Antibiotic prescribing for common infections in primary care in Pakistan, Egypt and Indonesia was very high, with antibiotics given in 70%–80% of patient consultations.
- Despite WHO AWaRe guidance recommending Access antibiotics or no antibiotics for most of these conditions, Watch antibiotics were frequently prescribed.
- High antibiotic prescribing appeared to be the norm, with low variability across prescribers, infections and specialisations.

**HOW THIS STUDY MIGHT AFFECT RESEARCH, PRACTICE OR POLICY**

- Urgent interventions are needed to address antibiotic overprescribing, which appears to have become routine in primary care and outpatient private healthcare in LMICs.

## Introduction

 Antibiotics play a major role in reducing the burden of bacterial infections. However, their success is threatened by increasing antimicrobial resistance (AMR).^1^ Overuse of antimicrobials is known to be a major driver of AMR in low and middle-income countries (LMICs).^2^ Yet, understanding the extent of antibiotic overuse in LMICs is often limited by lack of data on antibiotic prescribing.^3^ A paucity of data is particularly apparent in primary care and private settings, which serve as the formal first point of care for most patients with minor illnesses.^4^ These data gaps lead to challenges in conducting research, and potentially poor implementation of interventions to optimise antibiotic use.^5^

Pakistan, Egypt and Indonesia are three densely populated LMICs in the Eastern Mediterranean and South-East Asia regions with a significant burden of AMR. Previous studies based on aggregated data or small surveys suggest that antibiotic consumption in these countries is high and that antibiotics are being prescribed for conditions that generally do not require antibiotics. Antibiotic consumption in Pakistan is increased by 65% between 2000 and 2015, positioning this country as the fourth highest consumer of antibiotics among LMICs in 2015, when adjusted for population size. Indonesia experienced an estimated 2.5-fold increase in antibiotic consumption over the same period, largely driven by an increase in the use of broad-spectrum antibiotics.^6^,^8^ Although comparable statistics are not available for Egypt, a small-scale survey showed that 64% of prescribers prescribed antibiotics to treat colds and 21% acknowledged having prescribed antibiotics unnecessarily.^9^

While previous studies indicated that antibiotic overuse is common, a more comprehensive analysis on levels and patient-level and prescriber-level drivers of antibiotic prescribing practices are needed to generate the evidence base required for the design and implementation of effective antibiotic stewardship interventions. We, therefore, analysed data from large-scale prospectively collected surveys submitted by prescribers in 18 geographic regions of Pakistan, Egypt and Indonesia.

## Methods

### Data sources and procedures

We used three databases within the IQVIA Medical Data Index (Medical Index of Pakistan (MIP), Egypt Medical Data Index (EMDI), Indonesia Medical Data Index (IMDI)) for the period 2017–2021, which contain data on patient encounters prospectively collected by a representative sample of general practitioner and specialist prescribers, predominantly from the private sector, in Pakistan, Egypt and Indonesia. The study was designed to include prescribers primarily practising in the private sector; however, in settings where dual practice is common, outpatient prescriptions issued by the same prescribers in public facilities were anticipated and included. Data are collected in a standardised method across all countries.^10^

Prescribers were eligible if they were practising physicians contributing prescribing records during the study period. Prescribers practising exclusively in inpatient settings were not included. Participation was voluntary following explanation of the study. Prescribers were included using stratified random sampling. In each country, IQVIA established a sampling frame reflecting the national distribution of prescribers across regions and specialties. The sample size was determined by considering the number of prescribers and proportional distribution by geographic and specialty groups. For very uncommon specialities, proportional allocation may be insufficient; therefore, a minimum number of prescribers were recruited by IQVIA to ensure coverage. Operational feasibility considerations also informed the final sample size.

Information on proportional distribution of prescribers was updated annually by IQVIA; accordingly, target sample sizes could be modified from year to year to reflect changes in the underlying prescriber base and proportional distributions. Potential prescribers were then selected using systematic sampling with a random start from a mailing list of prescribers maintained by IQVIA, arranged in alphabetical order within the stratification criteria. Trained recruiters approached potential prescribers, explained the nature of the project and recruited participants until the target sample size was achieved. Recruitment was conducted evenly.

Data collection, which included patient encounters from all practice settings except hospital in-patient care, covered any reason for seeking medical care. Prescribers who agreed to participate were then instructed to record details of every patient encounter during a predetermined week each quarter or semester throughout the year. Information was recorded at the time of each encounter using structured paper-based casebooks provided by trained recruiters at the time of recruitment. The casebooks explained the completion procedure, included sections to record patient, diagnosis and treatment information for each encounter, contained a page on prescriber and practice characteristics and provided sufficient pages to capture all patient visits during the data collection period.

Prescribers reported data from all patient encounters, regardless of whether a prescription was issued. The data requested for entry included patient demographic information, diagnosis and treatment details (if any) in free text. IQVIA then coded diagnoses according to ICD-10 and classified prescribed medications using the European Pharmaceutical Research Association (EphMRA) Anatomical Therapeutic Classification (ATC) system.^11 12^ For all antibiotic products, we converted the EphMRA ATC codes to WHO ATC codes and added information on WHO AWaRe and Essential Medicines List (EML) classifications.^13 14^ Prescriber and practice characteristics, including prescriber’s sex, specialty and practice setting (hospital or clinic or others), were also collected from prescribers. Only aggregated data were transferred from IQVIA.

### Participants

We obtained health records for outpatient consultations related to common acute infections in Pakistan, Egypt and Indonesia between 2017 and 2021 (online supplemental appendix 1). All records were related to outpatient consultations in the private sector except in Indonesia, where 6.6% were from outpatient departments of government hospitals (table 1). These records were submitted by doctors across eight regions in Pakistan, four regions in Egypt and six regions in Indonesia. Visits were included if they had an ICD-10 code corresponding to an acute common infection diagnosis, falling within one of the following groups: respiratory tract infections (RTIs), gastrointestinal infections (GIs), urinary tract infections (UTIs), ear infections, skin infections or fever of unknown origin. The list of acute common infections was initially developed through a literature review of published research on similar topics.^15^ To ensure that no relevant acute infections were missed, we extracted diagnoses and ICD-10 codes from all visits associated with antibiotic prescriptions from the datasets. We added any conditions not initially included in the original list but deemed relevant on review into the final list (online supplemental appendix 2). The final list was reviewed by a panel comprising a general medical doctor, a pharmacist and an infectious disease specialist.

**Table 1:** Demographic and health-related characteristics of patients and prescribers by country

	Pakistan		Egypt		Indonesia	
	(n=204 822)		(n=87 286)		(n=92 807)	
Age, median (IQR))	25 (10–42)		26 (07–43)		27 (10–42)	
Age group						
≤ 5	31 153	(15.2)	19 738	(23)	14 417	(16)
6–10	15 152	(7.4)	7607	(8.7)	6499	(7)
10–17	21 054	(10.3)	6705	(7.7)	7169	(7.7)
18–24	28 728	(14)	8341	(9.6)	11 014	(12)
25–39	59 254	(28.4)	18 994	(22)	24 833	(27)
40–59	36 260	(17.7)	15 023	(17)	17 743	(19)
60–74	8325	(4.1)	4330	(5)	5372	(5.8)
≥ 75	4896	(2.4)	6450	(7.4)	4434	(4.8)
Unknown			98	(0.1)	1326	(1.4)
Sex, n (%)						
Male	109 349	(53.4)	45 331	(52)	44 774	(48)
Female	95 473	(46.6)	41 656	(48)	47 142	(51)
Unknown	–		299	(0.4)	891	(1)
Diagnosis, n (%)						
Respiratory tract infections:						
Acute upper respiratory tract infections	53 855	(26.3)	27 209	(31)	47 046	(51)
Acute lower respiratory tract infections	14 003	(6.8)	16 933	(19)	6767	(7.3)
Influenza	13 691	(6.7)	1465	(1.7)	1063	(1.2)
Other respiratory tract infections	28 600	(14)	1370	(1.6)	464	(0.5)
Other respiratory acute symptoms	46 482	(22.7)	5361	(6.1)	1369	(1.5)
Ear infections and mastoiditis	6691	(3.8)	7374	(8.5)	6610	(7.1)
Gastrointestinal infections	45 688	(22.3)	13 392	(15)	15 546	(17)
Urinary tract infections (UTIs)						
Cystitis	988	(0.5)	2309	(2.7)	730	(0.8)
Other complicated/unspecified UTIs	21 229	(10.4)	7035	(8.1)	5314	(5.7)
Skin infections	7036	(3.4)	5587	(6.4)	5848	(6.3)
Fever	59 936	(29.3)	7009	(8)	3966	(4.3)
Co-morbidities, n (%)						
Another acute infection diagnosis*	70 708	(34.5)	7381	(8.5)	2162	(2.3)
Other comorbidities†	85 307	(41.7)	13 112	(15)	13 188	(14)
Having a condition indicating severity, n (%)						
Accompanying fever‡	58 779	(28.7)	5804	(6.6)	3813	(4.1)
Disease-specific condition§	2834	(1.4)	1 097	(1.3)	325	(0.4)
Type of settings¶, n (%)						
Doctor’s clinic	125 656	(61.3)	76 967	(88)	38 042	(41)
Hospital	79 166	(38.7)	5064	(−5.8)	44 852	(48)
Other setting/unknown**	–		5255	(6)	9913	(11)
Prescriber’ specialisation						
General practitioner	125 358	(61.2)	14 816	(17)	50 948	(55)
Internal medicine	10 308	(5)	10 724	(12)	5689	(6.1)
Paediatrics	13 013	(6.4)	20 597	(24)	14 964	(16)
Otorhinolaryngology	7624	(3.7)	15 162	(17)	10 184	(11)
Pulmonology	8265	(4)	10 576	(12)	4030	(4.3)
Nephro-urology	–		4830	(5.5)	–	
Resident medical officers	24 988	(12.2)	–		–	
Dermatology	338	(0.7)	1473	(1.7)	1361	(1.5)
Surgery	2950	(1.4)	2972	(3.4)	1808	(2)
Others††	10 289	(5)	6 122	(7)	3003	(3.2)
Prescriber’s sex						
Male	188 233	(91.9)	78 405	(90)	40 011	(43)
Female	16 589	(8.1)	8881	(10)	52 796	(57)

Source: Author analysis of IQVIA Medical Data Index (Medical Index of Pakistan (MIP), Egypt Medical Data Index (EMDI), Indonesia Medical Data Index (IMDI)) for the period 2017–2021, reflecting estimates of real-world activity. Copyright IQVIA. All rights reserved.

* Another acute infection diagnosis includes cases with two or more of the acute infections listed above, excluding fever.

† Other comorbidities refer to cases where the patient was diagnosed with two or more conditions, excluding the acute infections listed above.

‡ Accompanying fever was counted for patients with fever alongside any of the acute infection diagnoses listed above, excluding fever itself.

§ Disease-specific conditions indicating severity include: shortness of breath, difficulty breathing or chest pain in patients with lower respiratory tract infections; bilateral ear pain, otorrhoea or ear discharge in patients with otitis; bloody diarrhoea in patients with gastrointestinal infections; blood in urine in patients with urinary tract infections; and swollen lymph nodes in patients with skin infections.

¶ In Pakistan and Egypt, all consultation settings were private. In Indonesia, the majority were private, with only 6.6% (16,840/92,807) conducted in public settings (government hospitals).

** Other settings included home visits, phone consultations and consultations where the setting was not specified.

†† Other specialisation included cardiology, rheumatology, gastroenterology/hepatology, endocrinology, ophthalmology, gynaecology, neurology, psychiatry, dentistry and oncology.

### Identification of antibiotic treatment

In this study, a consultation was considered to involve an antibiotic treatment if at least one systemic antibiotic was prescribed. We determined the antibiotic treatment status for each visit using the WHO AWaRe classification (categorised as Access, Watch or Reserve) and the WHO Model EML (categorised as EML or non-EML). For combinations classified as not-recommended or not included by WHO AWaRe, we categorised them based on the antibiotic with the ‘highest’ category within the combination (eg, a combination of Watch and Access antibiotics was classified as Watch). For single-agent antibiotics not listed in the WHO AWaRe classification, we assigned them to Access, Watch or Reserve categories by referencing the AWaRe classification of agents within the same chemical groups, typically at the same fourth level of the WHO ATC classification. When multiple antibiotics were used in a visit, the classification of the visit was based on the antibiotic with the highest WHO AWaRe category (ie, Reverse then Watch then Access). Visits with both a non-EML and EML antibiotic were classified as non-EML. We conducted this classification at the consultation level to ensure comparability between consultations in terms of Access, Watch and Reserve antibiotic use. Our classification of antibiotic treatment was conducted and reviewed by the panel above (online supplemental appendix 3).

### Statistical analyses

We conducted descriptive analyses of antibiotic prescribing practices, focusing on whether an antibiotic was prescribed and the type of antibiotic prescribed (categorised by WHO AWaRe and WHO EML) for health visits related to common infections in each country.

We analysed the determinants of the decision to prescribe an antibiotic or not (yes/no), and Watch antibiotic prescribing compared with Access antibiotics and a no-antibiotic strategy (Watch/Access/no antibiotic). We used mixed-effects multinomial regression models (mclogit package in R) to estimate ORs and 95% CIs. Variables included in the model were identified through a literature review of similar studies, considering the availability and accuracy of data on potential covariates in our dataset. Fixed effects included patient-specific factors (age; sex; clinical diagnosis; codiagnosis with other conditions; presence of accompanying clinical signs indicating severe infection) and prescriber-specific factors (sex of the prescriber, prescriber’s specialty and the type of clinical setting where the consultation occurred). Prescriber ID was included as a random effect to account for clustering by different health settings.

Statistical analysis was conducted using R V.4.4.1.

### Ethics

This study is a secondary data analysis using non-identifiable data from IQVIA’s Medical Data Index and, as such, does not require ethics approval.

### Patient and public involvement

Patients and the public were not involved in this research study.

## Results

Our analysis included a total of 384 975 consultation records from primary care and outpatient visits for acute common infections, with 204 882 records from Pakistan, 87 286 from Egypt and 92 807 from Indonesia. The median age of the patients was similar between the countries, ranging from 25 years (IQR 10–42) (Pakistan) to 27 years (IQR 10–42) (Indonesia). The most common infectious diagnoses were acute upper RTIs (Pakistan: 26.3%; Egypt: 31.3% and Indonesia: 50.7%) and GIs (Pakistan: 22.3%; Egypt: 13.4% and Indonesia: 16.8%).

Fever was more often documented in recorded consultations in Pakistan (29.3%) than in Egypt (8.0%) and Indonesia (4.3%). In Pakistan and Egypt, most records came from doctors’ clinics (61.4% and 88.2%, respectively). while in Indonesia, the distribution of records was more balanced across different types of settings. In each country, 80% of prescriptions were issued by prescribers specialising in one of the following fields: general practice or internal medicine, paediatrics, otorhinolaryngology or pulmonology (table 1).

### Antibiotic prescribing by country

Antibiotic treatment was prescribed in 82% of consultations in Pakistan, 81.2% in Egypt and 69.1% in Indonesia. Watch antibiotics accounted for 70.2% of all antibiotic prescriptions in Pakistan, 52.9% in Egypt and 53.6% in Indonesia. Access antibiotics were used less frequently (Pakistan: 24.2%, Egypt: 45.9% and Indonesia: 46.3%) and Reserve antibiotics were rarely used (Pakistan: 0.5%, Egypt: 1.2% and Indonesia: 0.1%). The use of non-EML antibiotics was also high, accounting for 26.8% of all antibiotic prescriptions in Pakistan, 39.9% in Egypt and 33.0% in Indonesia (table 2).

**Table 2:** Antibiotic prescribing patterns by infectious group and by country

	Antibiotic prescription	WHO AWaRe antibiotic prescription			WHO-EML antibiotic prescription	
	n (%)	Access	Watch	Reserve	EML	Non-EML
		n (%)	n (%)	n (%)	n (%)	n (%)
Pakistan						
All diagnoses combined (n=204 882)	167 957 (82)	49 473 (24.2)	117 952 (70.2)	3543 (0.5)	122 999 (73.2)	44 958 (26.8)
For specific diagnosis						
Respiratory tract infections:						
Acute upper respiratory tract infections	50 808 (94.3)	22 417 (44.1)	28 363 (55.8)	28 (0.1)	38 923 (76.6)	11 885 (23.4)
Acute lower respiratory tract infections	13 295 (94.9)	2599 (19.5)	10 565 (79.5)	131 (1)	8096 (60.9)	5199 (39.1)
Influenza	9551 (69.8)	3995 (41.8)	5553 (58.1)	3 (0)	7332 (76.8)	2219 (23.2)
Other respiratory tract infections	27 690 (96.8)	8900 (32.1)	18 745 (67.7)	45 (0.2)	19 935 (72)	7755 (28)
Other respiratory acute symptoms	33 113 (71.2)	11 455 (34.6)	21 622 (65.3)	36 (0.1)	24 760 (74.8)	8353 (25.2)
Ear infections and mastoiditis	6153 (92)	3007 (48.9)	3126 (50.8)	20 (0.3)	4346 (70.6)	1807 (29.4)
Gastrointestinal infections	38 719 (84.7)	6049 (15.6)	32 647 (84.3)	23 (0.1)	33 438 (86.4)	5281 (13.6)
Urinary tract infections (UTIs)						
Cystitis	913 (92.4)	110 (12.05)	803 (88)	0 (0)	557 (61)	356 (39)
Other complicated/unspecified UTIs	19 774 (93.1)	1264 (6.39)	18 472 (93.4)	38 (0.2)	10 752 (54.4)	9022 (45.6)
Skin infections	6547 (93.1)	4438 (67.79)	1860 (28.4)	249 (3.8)	3539 (54.1)	3008 (45.9)
Fever	45 477 (75.9)	15 703 (34.53)	29 741 (65.4)	33 (0.1)	35 312 (77.6)	10 165 (22.4)
Egypt						
All diagnoses combined (n=87 286)	70 841 (81.2)	32 540 (45.9)	37 441 (52.9)	860 (1.2)	42 613 (60.2)	28 228 (39.9)
For specific diagnosis						
Respiratory tract infections:						
Acute upper respiratory tract infections	23 121 (85)	14 417 (62.4)	8607 (37.2)	97 (0.4)	15 190 (65.7)	7931 (34.3)
Acute lower respiratory tract infections	15 474 (91.4)	4408 (28.5)	10 628 (68.7)	438 (2.8)	9550 (61.7)	5924 (38.3)
Influenza	945 (64.5)	463 (49)	472 (49.9)	10 (1.1)	654 (69.2)	291 (30.8)
Other respiratory tract infections	1269 (92.6)	326 (25.7)	904 (71.2)	39 (3.1)	761 (60)	508 (40)
Other respiratory acute symptoms	2314 (43.2)	1177 (50.9)	1129 (48.8)	8 (0.3)	1 642 (71)	672 (29)
Ear infections and mastoiditis	6270 (85)	3324 (53)	2934 (46.8)	12 (0.2)	4598 (73.3)	1672 (26.7)
Gastrointestinal infections	9428 (70.4)	3892 (41.3)	5485 (58.2)	51 (0.5)	3398 (36)	6030 (64)
UTIs						
Cystitis	2156 (93.4)	516 (23.9)	1637 (75.9)	3 (0.1)	1046 (48.5)	1110 (51.5)
Other complicated/unspecified UTIs	6436 (91.5)	1303 (20.2)	5100 (79.2)	33 (0.5)	3288 (51.1)	3148 (48.9)
Skin infections	5221 (93.4)	3533 (67.7)	1511 (28.9)	177 (3.4)	3586 (68.7)	1635 (31.3)
Fever	5236 (74.7)	2898 (55.3)	2317 (44.3)	21 (0.4)	3519 (67.2)	1717 (32.8)
Indonesia						
All diagnoses combined (n=92 807)	64 100 (69.1)	29 677 (46.3)	34 338 (53.6)	95 (0.1)	42 984 (67.1)	21 126 (33)
For specific diagnosis						
Respiratory tract infections:						
Acute upper respiratory tract infections	32 697 (69.5)	16 195 (49.5)	16 501 (50.5)	1 (0)	21 407 (65.5)	11 290 (34.5)
Acute lower respiratory tract infections	5470 (80.8)	1010 (18.5)	4445 (81.3)	15 (0.3)	3375 (61.7)	2095 (38.3)
Influenza	413 (38.9)	212 (51.3)	201 (48.7)	0 (0)	276 (66.8)	137 (33.2)
Other respiratory tract infections	226 (48.7)	62 (27.4)	164 (72.6)	0 (0)	126 (55.8)	100 (44.2)
Other respiratory acute symptoms	394 (28.8)	203 (51.5)	191 (48.5)	0 (0)	271 (68.8)	123 (31.2)
Ear infections and mastoiditis	4452 (67.4)	2226 (50)	2226 (50)	0 (0)	3448 (77.4)	1004 (22.6)
Gastrointestinal infections	9394 (60.4)	5945 (63.3)	3371 (35.9)	78 (0.8)	6442 (68.6)	2952 (31.4)
UTIs						
Cystitis	679 (93)	81 (11.9)	598 (88.1)	0 (0)	360 (53)	319 (47)
Other complicated/unspecified UTIs	5076 (95.5)	763 (15)	4313 (85)	0 (0)	3558 (70.1)	1518 (29.9)
Skin infections	4958 (84.8)	2580 (52)	2376 (47.9)	2 (0)	3578 (72.2)	1380 (27.8)
Fever	1843 (46.5)	1104 (59.9)	738 (40)	1 (0.1)	1216 (66)	627 (34)

Source: Author analysis of IQVIA Medical Data Index (Medical Index of Pakistan (MIP), Egypt Medical Data Index (EMDI), Indonesia Medical Data Index (IMDI)) for the period 2017–2021, reflecting estimates of real-world activity. Copyright IQVIA. All rights reserved.

EML, Essential Medicines List.

### Antibiotic prescribing by infection diagnosis

When the data were stratified by the 11 infection diagnoses, we found that antibiotic treatment was prescribed for over 80% of health consultations for most infection types in Pakistan (except influenza at 69.8% and other respiratory acute symptoms at 71.2%) and Egypt (except influenza at 64.5% and other acute respiratory symptoms at 43.2%). In Indonesia, where the overall antibiotic treatment rate was slightly lower (around 70% compared with 80% in Pakistan and Egypt), common infections like acute upper RTIs and GIs were still frequently treated with antibiotics (69.5% and 60.4%, respectively). Watch antibiotics were used in over 50% of antibiotic prescriptions for 10 out of 11 infections in Pakistan, 6 out of 11 in Egypt and 6 out of 11 in Indonesia (table 2).

**Figure 1:**
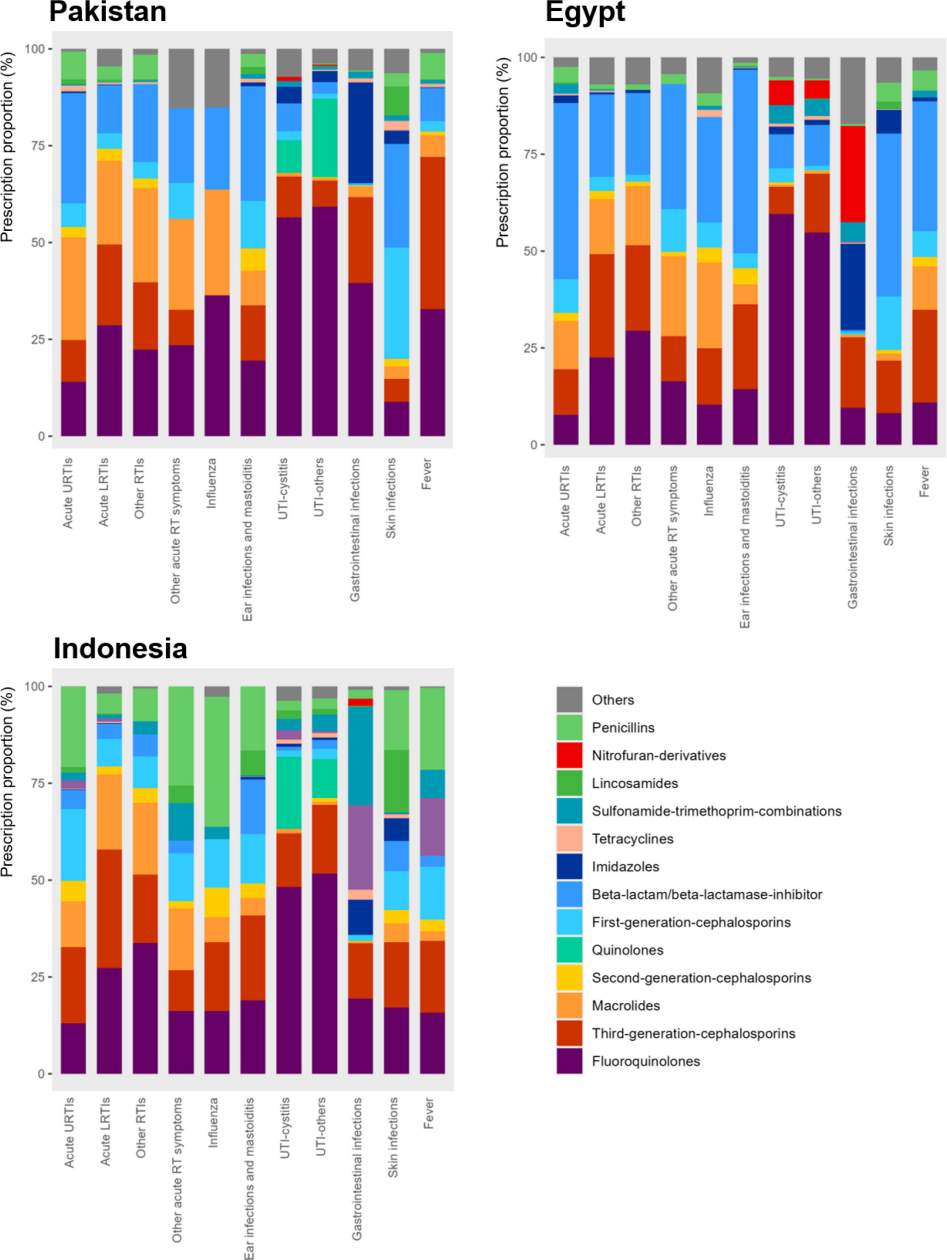
Distribution of antibiotic classes, by infectious group and by country. The pharmacological grouping follows the WHO AWaRe classification. https://www.who.int/publications/i/item/WHO-MHP-HPS-EML-2023.04 Source: Author analysis of IQVIA Medical Data Index (Medical Index of Pakistan (MIP), Egypt Medical Data Index (EMDI), Indonesia Medical Data Index (IMDI)) for the period 2017–2021, reflecting estimates of real-world activity. Copyright IQVIA. All rights reserved.

In all three countries, the most common pharmacological groups of antibiotics were those related to Watch antibiotics, including second/third-generation cephalosporins (mostly third-generation), quinolones and macrolides. Together, these three antibiotic classes accounted for 50%–75% of antibiotic prescriptions for most infections. The use of penicillins, such as amoxicillin, which are generally recommended as first-line Access antibiotics for most common infections,^16^ was very low (less than 5%) for most infections in Pakistan and Egypt. The use of penicillins was more common in Indonesia but remained below 25% of total antibiotic prescriptions for most infections (10/11, except for influenza, for which antibiotics are not recommended) (figure 1 and online supplemental appendix 4).

### Antibiotic prescribing by prescriber’s specialty

**Figure 2:**
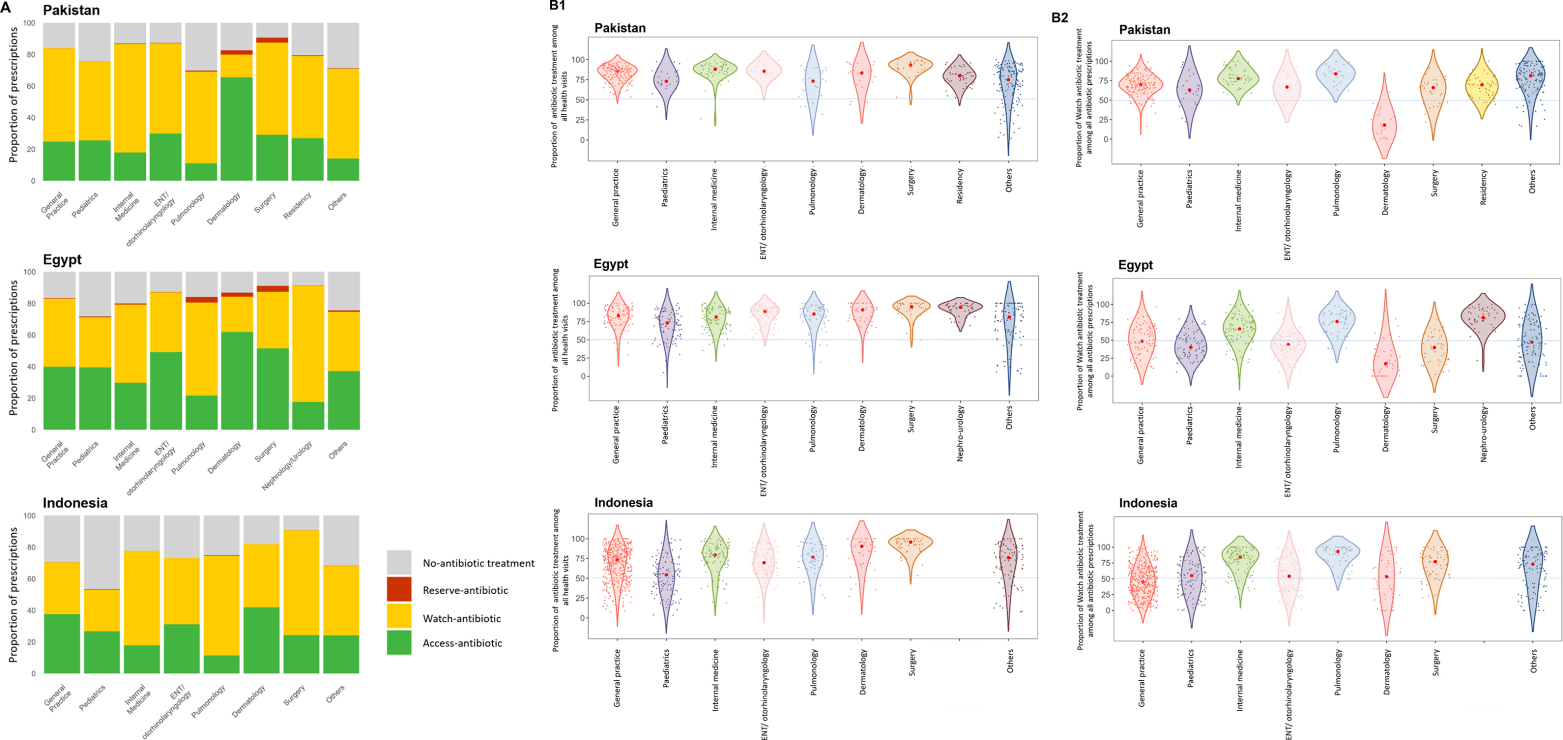
(A) Distributional proportions of WHO AWaRe antibiotic prescribing by prescriber’s speciality and by country. (B1) Variability in proportions of antibiotic prescriptions among doctors within and between specialities. (B2) Variability in proportions of Watch-antibiotic prescriptions relative to all antibiotic prescriptions among doctors within and between specialities. In Figures 2B1 and 2B2, the x-axis represents the specialities of prescribers who participated in the study in each country, while the y-axis represents the proportion of antibiotic prescriptions (2B1) and Watch-antibiotic prescriptions (2B2) by individual doctors, expressed as percentages ranging from 0% to 100%. Each dot corresponds to a specific doctor's prescribing proportion. Due to the kernel density estimation used in the violin plot, the visualisation may extend beyond this range, even though actual data points are confined within 0% to 100%. Source: Author analysis of IQVIA Medical Data Index (Medical Index of Pakistan (MIP), Egypt Medical Data Index (EMDI), Indonesia Medical Data Index (IMDI)) for the period 2017–2021, reflecting estimates of real-world activity. Copyright IQVIA. All rights reserved.

We observed consistently high antibiotic prescribing for common infections across most specialties, exceeding 75% in Pakistan and Egypt, and over 70% in Indonesia (figure 2A and online supplemental appendix 5). When grouping the data by prescriber, the median proportion of patients receiving any antibiotic prescription was high (above 75% in Pakistan and Egypt and above 60% in Indonesia) and the median proportions of prescribing Watch antibiotics were generally above 50% (figure 2B1-2). Variation in the proportion of consultations receiving any antibiotic or Watch antibiotic within the same specialty was least pronounced in Pakistan, higher in Egypt and most pronounced in Indonesia, as indicated by a lower spread in the antibiotic prescribing proportions among prescribers within the same specialty. For example, the IQR of the proportion of consultations receiving Watch antibiotics in general practices in Pakistan was 13.8%, compared with 28.2% in Egypt and 33.6% in Indonesia. This pattern was consistent across other medical specialties (figure 2B1-2 and online supplemental appendix 6).

### Determinants for antibiotic prescribing decisions

**Figure 3:**
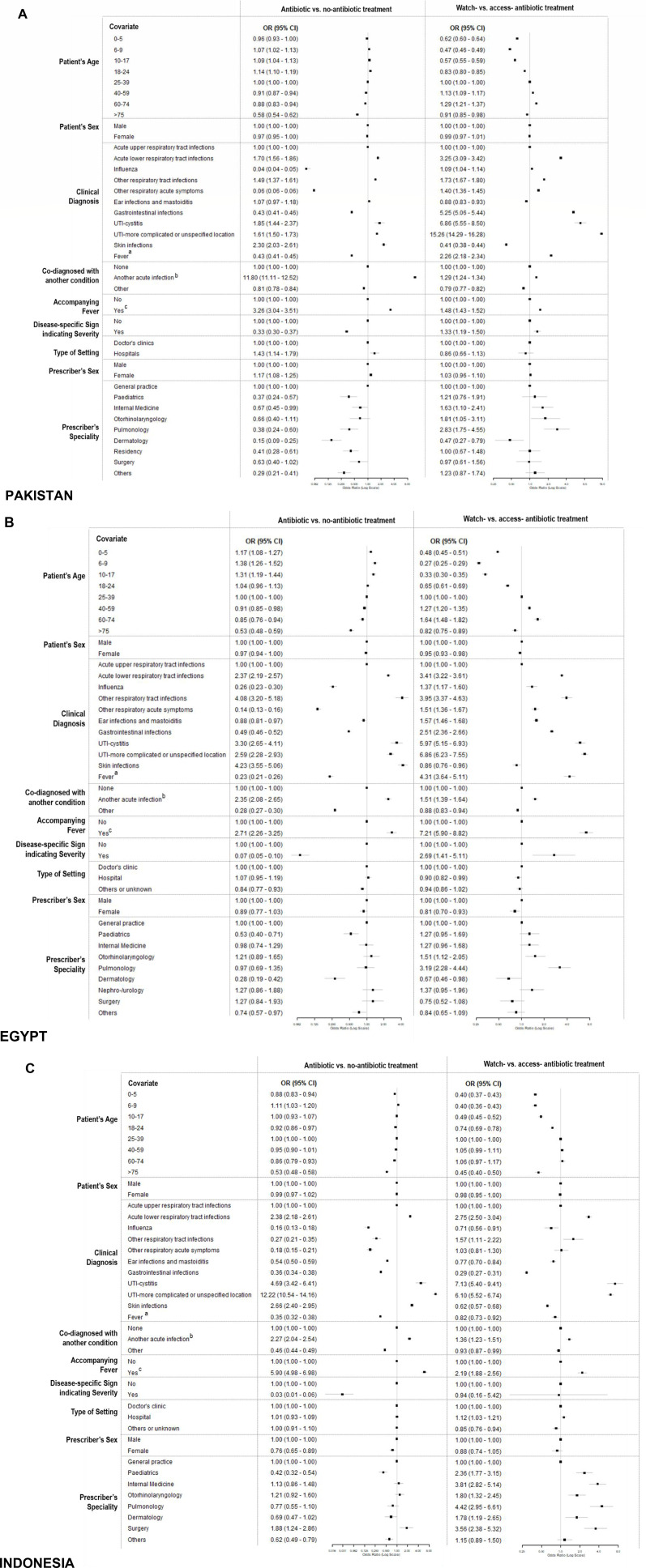
Factors associated with antibiotic prescribing decisions, including whether antibiotics were given and the choice between Watchantibiotics and Access-antibiotics, for patients in Pakistan (3A), Egypt (3B) and Indonesia (3C). a: cases with single diagnosis of fever, b: cases with more than one diagnosis of acute infections, c: cases with fever accompanying an acute infection. Source: Author analysis of IQVIA Medical Data Index (Medical Index of Pakistan (MIP), Egypt Medical Data Index (EMDI), Indonesia Medical Data Index (IMDI)) for the period 2017–2021, reflecting estimates of real-world activity. Copyright IQVIA. All rights reserved. UTIs, urinary tract infections.

In all recorded consultations across all three countries, increasing patient age was associated with a gradual reduction in the odds of prescribing any antibiotic, most notably in Egypt (figure 3A-C). Among consultations involving antibiotic treatment, consultations with patients aged 40–59 had higher odds of receiving Watch antibiotics over Access antibiotics (referred to as odds of receiving Watch antibiotics from now on) compared with those aged 25–39, while patients under 18 and over 75 had lower odds.

When compared with consultations for upper RTIs, those for lower RTIs, cystitis and other UTIs had the highest odds of including any antibiotics as well as Watch antibiotics. The adjusted ORs (aORs) for prescribing any antibiotics were: lower RTIs (Pakistan: 1.70, 95% CI 1.56 to 1.86; Egypt: 2.37, 2.19–2.57; Indonesia: 2.38, 2.18–2.61), cystitis (Pakistan: 1.85, 1.44–2.37; Egypt: 3.30, 2.65–4.11; Indonesia: 4.69, 3.42–6.41) and other UTIs (Pakistan: 1.61, 1.50–1.73; Egypt: 2.59, 2.28–2.93; Indonesia: 12.22, 10.54–14.16). The aORs for receiving Watch antibiotics were lower RTIs (Pakistan: 3.25, 3.09–3.42; Egypt: 3.41, 3.22–3.61; Indonesia: 2.75, 2.50–3.04), cystitis (Pakistan: 6.86, 5.55–8.50; Egypt: 5.97, 5.15–6.93; Indonesia: 7.13, 5.40–9.41) and other UTIs (Pakistan: 15.26, 14.29–16.28; Egypt: 6.86, 6.23–7.55; Indonesia: 6.10, 5.52–6.74). Several other conditions were associated with decreased odds of prescribing any antibiotics but elevated odds of prescribing Watch antibiotics, although this was not entirely consistent across all three countries. For example, in Pakistan, the aORs for prescribing any antibiotics in consultations for patients with GIs was 0.43 (0.41–0.46), while that for prescribing Watch antibiotics was 5.25 (5.06–5.44). While similar patterns were observed in Egypt, in Indonesia, patients with GIs were linked to decreased odds of prescribing both any (0.36, 0.34–0.38) and Watch (0.29, 0.27–0.31) antibiotics.

Compared with consultations for a single infection diagnosis, those with multiple acute infection diagnoses had elevated odds of prescribing any antibiotics (Pakistan: 11.80, 11.11–12.52; Egypt: 2.35, 2.08–2.65; Indonesia: 2.27, 2.04–2.95) or Watch antibiotics (Pakistan: 1.29, 1.24–1.34; Egypt: 1.51, 1.39–1.64; Indonesia: 1.36, 1.23–1.51). In contrast, consultations for patients codiagnosed with conditions outside of acute infections generally had reduced odds of prescribing antibiotics (eg, Pakistan: 0.81, 0.78–0.84 for any antibiotic prescription and 0.79, 0.77–0.82 for Watch antibiotic prescriptions).

Consultations for patients with an infection diagnosis and recorded fever had higher odds of prescribing any antibiotics (Pakistan: 3.26, 3.04–3.51; Egypt: 2.35, 2.08–2.65; Indonesia: 2.27, 2.04–2.95) or Watch antibiotics (Pakistan: 1.48, 1.43–1.52; Egypt: 2.35, 2.08–2.65; Indonesia: 2.27, 2.04–2.95) compared with those with an infectious diagnosis but no fever. In Pakistan and Egypt, consultations for patients with disease-specific signs indicating severity (eg, chest pain in patients with lower respiratory tract symptoms) had lower odds of prescribing antibiotics at the primary care level (Pakistan: 0.33, 0.30–0.37; Egypt: 0.07, 0.05–0.10), but if an antibiotic was prescribed, they had higher odds of prescribing a Watch antibiotic (Pakistan: 1.33, 1.19–1.50; Egypt: 2.69, 1.41–5.11). A similar trend was observed in Indonesia for any antibiotic prescription (0.03, 0.01–0.06), but there was no difference in the odds of prescribing Watch antibiotics (0.94, 0.16–5.42).

There was no substantial difference in the odds of prescribing either any antibiotics or Watch antibiotics between consultations at doctors’ clinics and hospitals across the three countries except that hospital clinic consultations in Pakistan had elevated odds of antibiotic treatments (1.43, 1.14–1.79). Consultations by female doctors in Indonesia had lower odds of including antibiotics (0.76, 0.65–0.89) and in Egypt had lower odds of including Watch antibiotics (0.81, 0.70–0.93). In contrast, consultations by female doctors in Pakistan were associated with higher odds of including antibiotics (1.17, 1.08–1.25). Compared with consultations by general practitioners, consultations by respiratory-related specialists in Pakistan (otorhinolaryngologists: 1.81, 1.05–3.11; pulmonologists: 2.83, 1.75–4.55) and Egypt (otorhinolaryngologists: 1.51, 1.12–2.05; pulmonologists: 3.19, 2.28–4.44) and with most specialists in Indonesia (paediatricians: 2.36, 1.77–3.15; internal medicine doctors: 3.81, 2.83–5.14; otorhinolaryngologists: 1.80, 1.32–2.45; pulmonologists: 4.42, 2.95–6.61; dermatologists: 1.78, 1.19–2.65; surgeons: 3.56, 2.38–5.32) had higher odds of prescribing Watch antibiotics.

## Discussion

Our findings highlight the high levels of antibiotic prescribing for primary care and outpatient consultations for common infections in private healthcare settings in Pakistan, Egypt and Indonesia. We observed antibiotic prescribing proportions as high as 70% to more than 80% of patient visits for common acute infections, including those that can generally be safely managed without antibiotics or for which antibiotics are not indicated, such as upper RTIs and viral influenza (see online supplemental appendix 7).^15^ Use of Watch antibiotics was common, despite the WHO AWaRe book guidance indicating that most infections evaluated here should be treated with Access antibiotics or no antibiotics at all.^13^

Our findings are consistent with observations from previous studies conducted in other LMICs. A narrative review by Saleem *et al* on AWARE antibiotic use in LMICs reported that private healthcare professionals prescribed antibiotics very commonly, and more frequently than those in the public sector.^17^ Lagarde and Duane reported that antibiotic prescribing for viral bronchitis was 66.7% in private clinics in Johannesburg, South Africa. Ingelbeen *et al* reported that 64.3% of visits to private clinics in Kongo Central province, Democratic Republic of Congo, resulted in an antibiotic prescription, compared with 48.8% in public primary care centres.^18 19^ Beri *et al* found that antibiotics were prescribed in 81% of cases of undifferentiated acute fever in Pune, India and that Watch-antibiotics, including oral fluoroquinolones, macrolides and cephalosporins, accounted for the largest proportions of prescribed antibiotic agents.^20^ Hassan *et al* used trained simulated patients presenting with common cold symptoms and reported antibiotic prescribing in 65% of encounters at private GP clinics in Penang, Malaysia.^21^ However, most of these studies were conducted on a smaller scale, focused on a single condition, and limited to specific regions.

Some of the common Watch antibiotic use may be due to the empiric treatment of suspected typhoid fever, which has a high incidence in Pakistan and Indonesia, and to a much lesser extent in Egypt.^22^ Watch antibiotics recommended for the treatment of uncomplicated typhoid fever in local guidelines in Pakistan were commonly used for patients with fever in our study (online supplemental appendix 4). Typhoid fever can be difficult to diagnose without diagnostic tests that are generally not widely available in LMICs, due to symptom overlap with infection types that often do not require antibiotics, such as signs of upper RTIs.^23^ Introduction of typhoid vaccines into routine immunisation programmes could remove this barrier to reductions in antibiotic use.^24^ To effectively reduce antibiotic prescribing in high-endemic countries, the vaccination programme should ideally be complemented by updated local prescribing guidance, training of prescribers and programmes raising the awareness among the general public to appropriately reflect changed pretest probabilities.^25^ Introducing a typhoid vaccination programme in the capital of Zimbabwe alone was, though, not associated with a reduction in antibiotic use the first year after the programme started.^25 26^

Patients presenting with fever were more likely to receive antibiotics, including Watch antibiotics. Fever is an important component of the FeverPAIN and Centor scores, which are commonly used to guide whether (Access) antibiotics are needed for patients presenting with a sore throat.^27^ Furthermore, when UTIs are accompanied by fever, the patient may have pyelonephritis for which the AWaRe guidance suggests a Watch antibiotic.

Reduced odds of being prescribed antibiotics in doctor visits for infections concurrently diagnosed with another non-infectious condition (compared with visits with a single infectious diagnosis) and for visits with disease-specific signs indicating severity (compared with those without such signs) could be explained by several mechanisms. First, those patients could be immediately admitted to hospital for antibiotic treatment. Second, some codiagnosed conditions or signs indicating severity are not specific for acute infections and might be attributed to underlying chronic conditions of the patients. For example, patients with acute cough and chest pain or shortness of breath due to underlying cardiovascular disease, or those with gastro-oesophageal reflux disease presenting with acute cough, may have a lower likelihood of being treated with antibiotics compared with patients with a single specific infectious diagnosis.^28 29^

Antibiotic prescribing decisions were not equal among all specialists, with adjusted odds of receiving Watch antibiotics being higher among patients treated by respiratory-related specialists in Pakistan and Egypt, and by most specialists in Indonesia. In LMICs, where specialists are involved in both primary and secondary care through their dual practice (ie, have a private clinic as well as a public facility role), they may drive the increased use of Watch antibiotics in the community by applying hospital-level prescribing practices, which typically empirically use more broad spectrum antibiotics for severe cases, to patients with minor infections.^30 31^ Beyond patient-related and prescriber-related factors, pharmaceutical suppliers may also play a role in Watch antibiotic overuse. The greatest number of Watch antibiotic products and suppliers was found in Pakistan (online supplemental appendix 8), the country with the highest proportion of Watch antibiotic prescriptions.^19^ This may increase pressure on prescribers to issue Watch antibiotics, although it could also reflect prescriber preferences within Pakistan’s private sector.

Overprescription of antibiotics for common infections in Pakistan, Egypt and Indonesia was documented previously.^24^,^26^ Overall, these studies reported either lower antibiotic prescribing proportions or a less predominant use of Watch antibiotics compared with our findings. However, most of the previous studies were conducted in public healthcare settings. In these settings, antibiotic prescribing is regulated by health authorities and public reimbursement agencies, which may reduce antibiotic overprescription. However, private clinics and hospitals are common—if not the most common—points of care for minor illnesses in these countries.^32^,^34^ With fewer regulatory restrictions, prescribing in private settings is largely influenced by prescriber preferences and patient demand, often driving antibiotic overuse. While our study focuses mainly on private settings, we found that outpatient consultations in private hospitals had higher odds of receiving Watch-antibiotics over Access-antibiotics than those in public hospitals in Indonesia (online supplemental appendix 9).

Among the three countries studied, Pakistan showed the highest antibiotic prescribing proportion, the most frequent use of Watch antibiotics, and the lowest variability on total antibiotic prescribing and Watch antibiotic prescribing proportions among prescribers within and across specialties. This low variability may indicate a strong social norm regarding antibiotic use among prescribers, which is a challenge when aiming to change prescribing behaviours.^35^ Previous studies have also emphasised the overuse and inappropriate use of antibiotics in Pakistan, alongside a culture of self-medication and purchasing antibiotics without prescriptions, all contributing to the high AMR burden in Pakistan.^1^

An important strength of this study is that it is one of the largest studies on antibiotic prescribing in LMICs, covering a wide range of infections and medical specialties while minimising recall bias and missing information on potential patient and prescriber-level characteristics through analysing data prospectively collected independent of antibiotic prescribing decisions. Our study is the largest to examine prescribing practices in private settings in LMICs. We also provided information on variability in prescribing practice among prescribers and identified, which factors may be driving antibiotic prescribing decisions.

This study has several limitations. The study sample may have limited generalisability due to a lack of coverage in certain regions within each included country and its focus on the private sector. As information on whether the healthcare settings were from rural or urban centres was not available, we were unable to compare antibiotic prescribing patterns between these areas. Doctors were required to report health records within only 1 week per quarter/semester during 2017–2020, which might be considered too short to fully represent their routine prescribing practices. Although the study protocol aimed to collect data on all health consultations, regardless of whether a prescription was issued, no visits without a prescription were recorded. This may indicate potential reporting bias, whereby consultations resulting in a prescription were more likely to be recorded, which could lead to an overestimation of antibiotic prescribing proportions. However, this complete capture of prescribing visits may also reflect patterns of care in private, out-of-pocket settings, where consultations frequently result in the provision of medicines and encounters without any drug therapy may be less commonly observed in routine practice. Patients with common infections in our sample may have been more severe, potentially leading to an overestimation of antibiotic overuse, as those with milder symptoms may go to drug stores instead of paying out-of-pocket for private care. It is possible that only prescribers confident in their prescribing practices agreed to participate in the surveys, causing an underestimation of the actual extent of antibiotic overuse. The self-reported prescription data may have reduced but not fully eliminated social desirability bias. Doctors might still report appropriate prescribing based on their perception, potentially underestimating antibiotic overprescribing. Our analysis on determinants of antibiotic prescribing might lack of critical factors, as not being recorded in the data collection. For example, the patient’s ability to pay, price of medications, patient’s demand and perceived needs of antibiotics.

In conclusion, high levels of antibiotic prescribing and predominant use of Watch antibiotics for common acute infections were observed across most infection types and prescribers in Pakistan, Egypt and Indonesia.

## Supplementary material

10.1136/bmjgh-2025-021139 online supplemental file 1

10.1136/bmjgh-2025-021139 online supplemental file 2

## Data Availability

Data may be obtained from a third party and are not publicly available.
