# Supplementary material for: AWaRe antibiotic prescribing for common acute infections in private primary care in low–middle-income countries: a patient-level analysis using IQVIA prescriber surveys from Pakistan, Egypt and Indonesia
Source: BMJ Glob Health. 2026 May 7;11(5):e021139. doi: 10.1136/bmjgh-2025-021139 (PMC13157772; doi:10.1136/bmjgh-2025-021139)
Supplement: online supplemental file 1 [file bmjgh-11-5-s001.docx]

### BMJ Global Health Author Reflexivity Statement

Adapted from Morton, B., Vercueil, A., Masekela, R., Heinz, E., Reimer, L., Saleh, S., Kalinga, C., Seekles, M., Biccard, B., Chakaya, J., Abimbola, S., Obasi, A. and Oriyo, N. (2022), Consensus statement on measures to promote equitable authorship in the publication of research from international partnerships. Anaesthesia, 77: 264-276. <https://doi.org/10.1111/anae.15597>

| **Study conceptualisation** | |
| --- | --- |
| 1. How does this study address local research and policy priorities? | This study addresses local research and policy priorities by providing large-scale evidence on antibiotic prescribing in primary care and outpatient settings in Pakistan, Egypt, and Indonesia, where high-quality data—especially from the private sector—are scarce. By documenting very high prescribing rates and frequent use of Watch antibiotics contrary to WHO AWaRe guidance, the findings highlight priority targets for antimicrobial stewardship. The results support national efforts to strengthen prescribing guidelines, surveillance, and interventions to reduce inappropriate antibiotic use and combat antimicrobial resistance. |
| 1. How were local researchers involved in study design? | This study used secondary data obtained from IQVIA, and the original survey design and data collection were determined by the data provider. The analytical plan for this study was developed specifically for the use of these data. Local researchers and clinical experts from the participating countries were invited to review the analytical approach, assess its appropriateness for the local healthcare context, and contribute to the interpretation of findings on antibiotic prescribing in Pakistan, Egypt, and Indonesia. |
| **Research management** | |
| 1. How has funding been used to support the local research team(s)? | Funding supported data access and the analytical work for this study and facilitated collaboration with researchers and clinical experts from the participating countries. This engagement helped ensure appropriate analysis, contextual interpretation, and locally relevant dissemination of the findings. |
| **Data acquisition and analysis** | |
| 1. How are research staff who conducted data collection acknowledged? | This study used secondary data obtained from IQVIA, and data collection was conducted by the data provider according to their established procedures. The contribution of IQVIA in generating and maintaining the dataset is acknowledged in the manuscript. |
| 1. How have members of the research partnership been provided with access to study data? | Access to the study data was managed in accordance with the data-use agreement with IQVIA. The dataset was accessed by authorised members of the study team for analysis, and results and analytical outputs were shared within the team to support collaborative review and interpretation while maintaining compliance with data governance requirements. |
| 1. How were data used to develop analytical skills within the partnership? | The study involved collaborative development and review of the analytical approach within the study team, including discussions on variable definitions, modelling strategies, and interpretation of results. This process supported shared understanding of the dataset and analytical methods and facilitated knowledge exchange among team members. |
| **Data interpretation** | |
| 1. How have research partners collaborated in interpreting study data? | Interpretation of the study findings involved collaborative review within the study team, with input from clinical experts familiar with antibiotic prescribing practices and healthcare systems in the participating countries. Their contextual insights helped assess the plausibility of results and supported interpretation of prescribing patterns within local settings. |
| **Drafting and revising for intellectual content** | |
| 1. How were research partners supported to develop writing skills? | Manuscript drafting and revision were undertaken collaboratively within the study team, with contributors providing input on interpretation, clinical context, and relevance to local healthcare settings. This collaborative writing process enabled exchange of perspectives and supported clear communication of the findings. |
| 1. How will research products be shared to address local needs? | Study findings will be disseminated through peer-reviewed publication and academic presentations, with emphasis on communicating results relevant to antibiotic prescribing and antimicrobial stewardship in the participating countries. The outputs are intended to inform researchers, clinicians, and policymakers and support efforts to address inappropriate antibiotic use in local healthcare settings. |
| **Authorship** | |
| 1. How is the leadership, contribution and ownership of this work by LMIC researchers recognised within the authorship? | Experts with knowledge of the participating countries contributed to the analytical approach and interpretation of findings. Authorship was assigned according to standard criteria, recognising substantive intellectual contributions and relevant expertise. |
| 1. How have early career researchers across the partnership been included within the authorship team? | Early career researchers contributed to analysis, interpretation, and manuscript preparation and were included as authors where they met standard authorship criteria. |
| 1. How has gender balance been addressed within the authorship? | Authorship was determined based on expertise and substantive contribution, with attention to gender diversity and inclusive representation within the team. |
| **Training** | |
| 1. How has the project contributed to training of LMIC researchers? | The project involved engagement with researchers and clinical experts from the LMIC study settings in reviewing the analytical approach and interpreting findings. This process supported knowledge exchange and familiarisation with the study methods and results relevant to antibiotic prescribing in their local healthcare contexts. |
| **Infrastructure** | |
| 1. How has the project contributed to improvements in local infrastructure? | The project did not involve direct investment in physical infrastructure. However, by generating standardised evidence on antibiotic prescribing patterns in the participating LMIC settings, the study contributes to strengthening the evidence base that can support surveillance, stewardship planning, and data-informed decision-making within local health systems. |
| **Governance** | |
| 1. What safeguarding procedures were used to protect local study participants and researchers? | The study used anonymised secondary data held and managed by IQVIA. The research team did not directly access identifiable or raw individual-level data; analytical code was developed using dummy data and executed by the data provider within their secure environment. This approach ensured confidentiality of participants and compliance with applicable data governance and security procedures. |
