# Supplementary material for: AWaRe antibiotic prescribing for common acute infections in private primary care in low–middle-income countries: a patient-level analysis using IQVIA prescriber surveys from Pakistan, Egypt and Indonesia
Source: BMJ Glob Health. 2026 May 7;11(5):e021139. doi: 10.1136/bmjgh-2025-021139 (PMC13157772; doi:10.1136/bmjgh-2025-021139)
Supplement: online supplemental file 2 [file bmjgh-11-5-s002.docx]

**Appendix 1.** The total number of doctors submitting records of health consultations (for any visits or visits related to common conditions) and the total number of records of health consultations submitted (for any visits or common conditions) were recorded per quarter or term in each country: A) Pakistan, B) Egypt, C) Indonesia

Source: Author analysis of IQVIA Medical Data Index (Medical Index of Pakistan (MIP), Egypt Medical Data Index (EMDI), Indonesia Medical Data Index (IMDI)) for the period 2017-2021, reflecting estimates of real world activity. Copyright IQVIA. All rights reserved.

1. **PAKISTAN**

| Region | Quarter/  Term | Year | Total number of doctors submitting records of health consultations for any condition | Number of records of health consultations submitted | Number of doctors submitting records of health consultations for common infection-related visits | Number of records of health consultations for common infection-related visits |
| --- | --- | --- | --- | --- | --- | --- |
| Baluchistan | 1 | 2017 | 19 | 1931 | 16 | 700 |
| Baluchistan | 2 | 2017 | 19 | 1952 | 15 | 700 |
| Baluchistan | 1 | 2018 | 19 | 1961 | 16 | 867 |
| Baluchistan | 2 | 2018 | 19 | 2040 | 16 | 769 |
| Baluchistan | 1 | 2019 | 19 | 2078 | 15 | 759 |
| Baluchistan | 2 | 2019 | 19 | 2204 | 17 | 790 |
| Baluchistan | 1 | 2020 | 19 | 2084 | 15 | 785 |
| Baluchistan | 2 | 2020 | 19 | 2140 | 16 | 819 |
| Faisalabad | 1 | 2017 | 41 | 5415 | 40 | 2117 |
| Faisalabad | 2 | 2017 | 41 | 5739 | 40 | 2141 |
| Faisalabad | 1 | 2018 | 41 | 5807 | 38 | 2133 |
| Faisalabad | 2 | 2018 | 41 | 5937 | 36 | 2151 |
| Faisalabad | 1 | 2019 | 41 | 6113 | 38 | 2372 |
| Faisalabad | 2 | 2019 | 41 | 6159 | 39 | 2536 |
| Faisalabad | 1 | 2020 | 40 | 5616 | 37 | 2293 |
| Faisalabad | 2 | 2020 | 42 | 6377 | 39 | 2362 |
| Karachi | 1 | 2017 | 137 | 18409 | 120 | 6501 |
| Karachi | 2 | 2017 | 137 | 18668 | 121 | 6489 |
| Karachi | 1 | 2018 | 138 | 19053 | 123 | 6708 |
| Karachi | 2 | 2018 | 138 | 19436 | 124 | 6554 |
| Karachi | 1 | 2019 | 138 | 19956 | 121 | 7331 |
| Karachi | 2 | 2019 | 137 | 19680 | 117 | 6761 |
| Karachi | 1 | 2020 | 133 | 19150 | 112 | 6614 |
| Karachi | 2 | 2020 | 137 | 19410 | 120 | 6771 |
| Lahore | 1 | 2017 | 129 | 17419 | 107 | 4621 |
| Lahore | 2 | 2017 | 129 | 17879 | 105 | 4629 |
| Lahore | 1 | 2018 | 129 | 17931 | 107 | 4703 |
| Lahore | 2 | 2018 | 129 | 18282 | 107 | 4915 |
| Lahore | 1 | 2019 | 129 | 18319 | 109 | 5176 |
| Lahore | 2 | 2019 | 128 | 18408 | 105 | 5047 |
| Lahore | 1 | 2020 | 126 | 17439 | 101 | 4869 |
| Lahore | 2 | 2020 | 127 | 18123 | 101 | 5003 |
| Multan | 1 | 2017 | 47 | 7160 | 40 | 2702 |
| Multan | 2 | 2017 | 47 | 7299 | 39 | 2779 |
| Multan | 1 | 2018 | 46 | 7103 | 38 | 2546 |
| Multan | 2 | 2018 | 46 | 7129 | 38 | 2573 |
| Multan | 1 | 2019 | 46 | 7276 | 39 | 2630 |
| Multan | 2 | 2019 | 47 | 7738 | 43 | 2723 |
| Multan | 1 | 2020 | 47 | 7630 | 38 | 2843 |
| Multan | 2 | 2020 | 47 | 7787 | 40 | 2912 |
| Peshawar | 1 | 2017 | 46 | 5486 | 40 | 1663 |
| Peshawar | 2 | 2017 | 46 | 5891 | 42 | 1761 |
| Peshawar | 1 | 2018 | 46 | 5964 | 41 | 1723 |
| Peshawar | 2 | 2018 | 46 | 6142 | 43 | 1958 |
| Peshawar | 1 | 2019 | 46 | 6256 | 44 | 2022 |
| Peshawar | 2 | 2019 | 46 | 6487 | 42 | 2111 |
| Peshawar | 1 | 2020 | 46 | 6363 | 42 | 2081 |
| Peshawar | 2 | 2020 | 46 | 6335 | 44 | 1965 |
| Rawalpindi | 1 | 2017 | 63 | 8048 | 56 | 2248 |
| Rawalpindi | 2 | 2017 | 63 | 8217 | 55 | 2597 |
| Rawalpindi | 1 | 2018 | 63 | 8312 | 55 | 2719 |
| Rawalpindi | 2 | 2018 | 63 | 8457 | 57 | 2935 |
| Rawalpindi | 1 | 2019 | 63 | 8655 | 56 | 2855 |
| Rawalpindi | 2 | 2019 | 64 | 8953 | 55 | 3026 |
| Rawalpindi | 1 | 2020 | 64 | 9007 | 56 | 2948 |
| Rawalpindi | 2 | 2020 | 64 | 9051 | 55 | 3008 |
| Sindh | 1 | 2017 | 58 | 8799 | 55 | 3485 |
| Sindh | 2 | 2017 | 58 | 8871 | 53 | 3519 |
| Sindh | 1 | 2018 | 58 | 8932 | 53 | 3610 |
| Sindh | 2 | 2018 | 58 | 9018 | 53 | 3556 |
| Sindh | 1 | 2019 | 58 | 9058 | 52 | 3535 |
| Sindh | 2 | 2019 | 58 | 9190 | 54 | 3564 |
| Sindh | 1 | 2020 | 56 | 8930 | 51 | 3605 |
| Sindh | 2 | 2020 | 58 | 9225 | 53 | 3634 |

1. **EGYPT**

| Region | Quarter/  Term | Year | Total number of doctors submitting records of health consultations for any condition | Number of records of health consultations submitted | Number of doctors submitting records of health consultations for common infection-related visits | Number of records of health consultations for common infection-related visits |
| --- | --- | --- | --- | --- | --- | --- |
| Alexandria | 1 | 2017 | 83 | 3111 | 58 | 683 |
| Alexandria | 2 | 2017 | 83 | 3059 | 58 | 654 |
| Alexandria | 3 | 2017 | 86 | 3321 | 61 | 683 |
| Alexandria | 4 | 2017 | 86 | 3248 | 65 | 689 |
| Alexandria | 1 | 2018 | 86 | 1848 | 57 | 378 |
| Alexandria | 2 | 2018 | 86 | 3249 | 62 | 674 |
| Alexandria | 3 | 2018 | 86 | 3267 | 64 | 684 |
| Alexandria | 4 | 2018 | 86 | 3550 | 64 | 743 |
| Alexandria | 1 | 2019 | 86 | 3489 | 65 | 743 |
| Alexandria | 2 | 2019 | 86 | 3496 | 66 | 717 |
| Alexandria | 3 | 2019 | 85 | 3398 | 54 | 704 |
| Alexandria | 4 | 2019 | 85 | 3333 | 58 | 619 |
| Alexandria | 1 | 2020 | 85 | 2923 | 55 | 534 |
| Alexandria | 2 | 2020 | 85 | 2594 | 54 | 472 |
| Alexandria | 3 | 2020 | 85 | 2664 | 49 | 460 |
| Alexandria | 4 | 2020 | 85 | 3056 | 52 | 552 |
| Alexandria | 1 | 2021 | 85 | 3150 | 50 | 512 |
| Alexandria | 2 | 2021 | 85 | 3325 | 53 | 577 |
| Alexandria | 3 | 2021 | 85 | 3245 | 51 | 576 |
| Cariro - Giza | 1 | 2017 | 211 | 9116 | 148 | 1854 |
| Cariro - Giza | 2 | 2017 | 213 | 9277 | 150 | 1839 |
| Cariro - Giza | 3 | 2017 | 222 | 9287 | 154 | 1752 |
| Cariro - Giza | 4 | 2017 | 224 | 9425 | 157 | 1809 |
| Cariro - Giza | 1 | 2018 | 222 | 4818 | 126 | 992 |
| Cariro - Giza | 2 | 2018 | 224 | 9537 | 157 | 1834 |
| Cariro - Giza | 3 | 2018 | 223 | 9363 | 154 | 1697 |
| Cariro - Giza | 4 | 2018 | 224 | 10026 | 157 | 1911 |
| Cariro - Giza | 1 | 2019 | 224 | 10204 | 159 | 1964 |
| Cariro - Giza | 2 | 2019 | 224 | 10330 | 152 | 1977 |
| Cariro - Giza | 3 | 2019 | 253 | 11672 | 171 | 2117 |
| Cariro - Giza | 4 | 2019 | 253 | 10707 | 175 | 1922 |
| Cariro - Giza | 1 | 2020 | 253 | 9672 | 177 | 1776 |
| Cariro - Giza | 2 | 2020 | 251 | 7495 | 159 | 1265 |
| Cariro - Giza | 3 | 2020 | 252 | 8020 | 165 | 1225 |
| Cariro - Giza | 4 | 2020 | 253 | 9650 | 159 | 1553 |
| Cariro - Giza | 1 | 2021 | 253 | 9474 | 157 | 1370 |
| Cariro - Giza | 2 | 2021 | 252 | 9607 | 156 | 1423 |
| Cariro - Giza | 3 | 2021 | 253 | 9365 | 161 | 1300 |
| Delta/Canal Zone | 1 | 2017 | 145 | 5722 | 107 | 1261 |
| Delta/Canal Zone | 2 | 2017 | 145 | 5759 | 109 | 1243 |
| Delta/Canal Zone | 3 | 2017 | 148 | 5980 | 111 | 1243 |
| Delta/Canal Zone | 4 | 2017 | 148 | 6056 | 113 | 1311 |
| Delta/Canal Zone | 1 | 2018 | 147 | 3208 | 89 | 726 |
| Delta/Canal Zone | 2 | 2018 | 148 | 5938 | 99 | 1187 |
| Delta/Canal Zone | 3 | 2018 | 148 | 6139 | 106 | 1220 |
| Delta/Canal Zone | 4 | 2018 | 148 | 6304 | 106 | 1317 |
| Delta/Canal Zone | 1 | 2019 | 148 | 6352 | 103 | 1274 |
| Delta/Canal Zone | 2 | 2019 | 148 | 6398 | 105 | 1331 |
| Delta/Canal Zone | 3 | 2019 | 207 | 8565 | 154 | 1796 |
| Delta/Canal Zone | 4 | 2019 | 206 | 8157 | 151 | 1751 |
| Delta/Canal Zone | 1 | 2020 | 207 | 7653 | 144 | 1541 |
| Delta/Canal Zone | 2 | 2020 | 207 | 6412 | 141 | 1314 |
| Delta/Canal Zone | 3 | 2020 | 207 | 6379 | 143 | 1189 |
| Delta/Canal Zone | 4 | 2020 | 207 | 7786 | 148 | 1457 |
| Delta/Canal Zone | 1 | 2021 | 207 | 8089 | 145 | 1355 |
| Delta/Canal Zone | 2 | 2021 | 207 | 8371 | 147 | 1452 |
| Delta/Canal Zone | 3 | 2021 | 207 | 8575 | 151 | 1522 |
| Upper Egypt | 1 | 2017 | 84 | 3683 | 66 | 949 |
| Upper Egypt | 2 | 2017 | 84 | 3816 | 65 | 996 |
| Upper Egypt | 3 | 2017 | 87 | 4012 | 66 | 953 |
| Upper Egypt | 4 | 2017 | 87 | 3923 | 63 | 920 |
| Upper Egypt | 1 | 2018 | 87 | 2259 | 60 | 547 |
| Upper Egypt | 2 | 2018 | 87 | 3762 | 62 | 910 |
| Upper Egypt | 3 | 2018 | 87 | 3840 | 64 | 846 |
| Upper Egypt | 4 | 2018 | 87 | 4077 | 64 | 977 |
| Upper Egypt | 1 | 2019 | 87 | 4025 | 66 | 938 |
| Upper Egypt | 2 | 2019 | 87 | 4088 | 66 | 969 |
| Upper Egypt | 3 | 2019 | 110 | 5199 | 81 | 1279 |
| Upper Egypt | 4 | 2019 | 110 | 5287 | 81 | 1234 |
| Upper Egypt | 1 | 2020 | 110 | 4583 | 82 | 1009 |
| Upper Egypt | 2 | 2020 | 110 | 4145 | 80 | 870 |
| Upper Egypt | 3 | 2020 | 110 | 4420 | 82 | 899 |
| Upper Egypt | 4 | 2020 | 110 | 4930 | 80 | 1088 |
| Upper Egypt | 1 | 2021 | 110 | 5081 | 78 | 1024 |
| Upper Egypt | 2 | 2021 | 110 | 5294 | 78 | 1083 |
| Upper Egypt | 3 | 2021 | 110 | 5308 | 80 | 1071 |

1. **INDONESIA**

| Region | Quarter/  Term | Year | Total number of doctors submitting records of health consultations for any condition | Number of records of health consultations submitted | Number of doctors submitting records of health consultations for common infection-related visits | Number of records of health consultations for common infection-related visits |
| --- | --- | --- | --- | --- | --- | --- |
| Central Java | 1 | 2017 | 129 | 11461 | 104 | 2244 |
| Central Java | 2 | 2017 | 124 | 11200 | 103 | 2130 |
| Central Java | 1 | 2018 | 122 | 10986 | 103 | 2096 |
| Central Java | 2 | 2018 | 115 | 10348 | 91 | 1999 |
| Central Java | 1 | 2019 | 120 | 11195 | 98 | 2247 |
| East Indonesia | 1 | 2017 | 85 | 8064 | 75 | 1983 |
| East Indonesia | 2 | 2017 | 84 | 8125 | 75 | 1949 |
| East Indonesia | 1 | 2018 | 77 | 7339 | 64 | 1929 |
| East Indonesia | 2 | 2018 | 91 | 8770 | 82 | 2353 |
| East Indonesia | 1 | 2019 | 96 | 9327 | 86 | 2508 |
| East Java | 1 | 2017 | 135 | 11304 | 103 | 2154 |
| East Java | 2 | 2017 | 136 | 12710 | 97 | 2373 |
| East Java | 1 | 2018 | 140 | 13028 | 103 | 2515 |
| East Java | 2 | 2018 | 134 | 12583 | 98 | 2107 |
| East Java | 1 | 2019 | 133 | 12622 | 99 | 2377 |
| Jakarta | 1 | 2017 | 259 | 24223 | 199 | 4964 |
| Jakarta | 2 | 2017 | 266 | 25095 | 211 | 5100 |
| Jakarta | 1 | 2018 | 280 | 26426 | 219 | 5130 |
| Jakarta | 2 | 2018 | 280 | 26704 | 209 | 5183 |
| Jakarta | 1 | 2019 | 276 | 26572 | 210 | 5655 |
| Sumatra | 1 | 2017 | 180 | 17307 | 154 | 3615 |
| Sumatra | 2 | 2017 | 167 | 16167 | 134 | 3381 |
| Sumatra | 1 | 2018 | 167 | 16105 | 137 | 3314 |
| Sumatra | 2 | 2018 | 171 | 16444 | 140 | 3243 |
| Sumatra | 1 | 2019 | 170 | 16475 | 136 | 3263 |
| West Java | 1 | 2017 | 156 | 14487 | 121 | 3379 |
| West Java | 2 | 2017 | 160 | 15010 | 130 | 3471 |
| West Java | 1 | 2018 | 159 | 14832 | 125 | 3531 |
| West Java | 2 | 2018 | 148 | 13839 | 116 | 3366 |
| West Java | 1 | 2019 | 138 | 13007 | 109 | 3248 |

**Appendix 2.** List of ICD-10 code corresponding to common acute infection diagnoses included in the study

| **ICD code** | **Diagnosis** | **Diagnosis groups** |
| --- | --- | --- |
| R50 | Fever of other and unknown origin | Fever |
| A00 | Cholera | Gastrointestinal infections |
| A01 | Typhoid and paratyphoid fevers | Gastrointestinal infections |
| A02 | Other salmonella infections | Gastrointestinal infections |
| A03 | Shigellosis | Gastrointestinal infections |
| A04 | Other bacterial intestinal infections | Gastrointestinal infections |
| A05 | Other bacterial foodborne intoxications, not elsewhere classified | Gastrointestinal infections |
| A06 | Amoebiasis | Gastrointestinal infections |
| A07 | Other protozoal intestinal diseases | Gastrointestinal infections |
| A08 | Viral and other specified intestinal infections | Gastrointestinal infections |
| A09 | Other gastroenteritis and colitis of infectious and unspecified origin | Gastrointestinal infections |
| H60.0 | Abscess of external ear | Mastoiditis and other ear infection complications |
| H60.1 | Cellulitis of external ear | Mastoiditis and other ear infection complications |
| H60.2 | Malignant otitis externa | Mastoiditis and other ear infection complications |
| H60.8 | Other otitis externa | Mastoiditis and other ear infection complications |
| H60.9 | Otitis externa, unspecified | Mastoiditis and other ear infection complications |
| H60.3 | Other infective otitis externa | Mastoiditis and other ear infection complications |
| H62.0 | Otitis externa in bacterial diseases classified elsewhere | Mastoiditis and other ear infection complications |
| H62.3 | Otitis externa in other infectious and parasitic diseases classified elsewhere | Mastoiditis and other ear infection complications |
| H62.4 | Otitis externa in other diseases classified elsewhere | Mastoiditis and other ear infection complications |
| H65.0 | Acute serious otitis media | Mastoiditis and other ear infection complications |
| H65.1 | Other acute nonsuppurative otitis media | Mastoiditis and other ear infection complications |
| H65.9 | Nonsuppurative otitis media, unspecified | Mastoiditis and other ear infection complications |
| H66.0 | Acute suppurative otitis media | Mastoiditis and other ear infection complications |
| H66.4 | Suppurative otitis media, unspecified | Mastoiditis and other ear infection complications |
| H66.9 | Otitis media, unspecified | Mastoiditis and other ear infection complications |
| H67.0 | Otitis media, unspecified | Mastoiditis and other ear infection complications |
| H67.8 | Otitis media in bacterial diseases classified elsewhere | Mastoiditis and other ear infection complications |
| H70 | Mastoiditis and related conditions | Mastoiditis and other ear infection complications |
| H74 | Other disorders of middle ear mastoid | Mastoiditis and other ear infection complications |
| H75 | Other disorders of middle ear and mastoid in diseases classified elsewhere | Mastoiditis and other ear infection complications |
| J12 | Viral pneumonia, not elsewhere classified | Acute lower RTIs |
| J13 | Pneumonia due to Streptococcus pneumoniae | Acute lower RTIs |
| J14 | Pneumonia due to Haemophilus influenzae | Acute lower RTIs |
| J15 | Bacterial pneumonia, not elsewhere classified | Acute lower RTIs |
| J16 | Pneumonia due to other infectious organisms, not elsewhere classified | Acute lower RTIs |
| J17 | Pneumonia in diseases classified elsewhere | Acute lower RTIs |
| J18 | Pneumonia, organism unspecified | Acute lower RTIs |
| J20 | Acute bronchitis | Acute lower RTIs |
| J21 | Acute bronchiolitis | Acute lower RTIs |
| J22 | Unspecified acute lower respiratory infection | Acute lower RTIs |
| J44.0 | COPD with acute lower respiratory tract infection | Acute lower RTIs |
| J40 | Bronchitis, not specified as acute or chronic | Acute lower RTIs |
| J44.1 | COPD with acute exacerbation, unspecified | Acute lower RTIs |
| A36.0 | Pharyngeal diphtheria | Acute upper RTIs |
| A36.1 | Nasopharyngeal diphtheria | Acute upper RTIs |
| A36.2 | Laryngeal diphtheria | Acute upper RTIs |
| J00 | Acute nasopharyngitis | Acute upper RTIs |
| J01 | Acute sinusitis | Acute upper RTIs |
| J02 | Acute pharyngitis | Acute upper RTIs |
| J03 | Acute tonsillitis | Acute upper RTIs |
| J04 | Acute laryngitis and tracheitis | Acute upper RTIs |
| J05 | Acute obstructive laryngitis [croup] and epiglottitis | Acute upper RTIs |
| J06 | Acute upper respiratory infections of multiple and unspecified sites | Acute upper RTIs |
| J36 | Peritonsillar abscess | Acute upper RTIs |
| J39.0 | Retropharyngeal and parapharyngeal abscess | Acute upper RTIs |
| J39.1 | Other abscess of pharynx | Acute upper RTIs |
| J10 | Influenza due to identified seasonal influenza virus | Influenza |
| J11 | Influenza, virus not identified | Influenza |
| R05 | Cough | Other respiratory acute symptoms |
| R07.0 | Pain in throat | Other respiratory acute symptoms |
| R07.3 | Chest pain on breathing | Other respiratory acute symptoms |
| R07.4 | Chest pain, unspecified | Other respiratory acute symptoms |
| R09.3 | Abnormal sputum | Other respiratory acute symptoms |
| J98.7 | Respiratory infections, not elsewhere classified (Respiratory (tract) infections not specified as acute, chronic, lower, or upper) | Other RTIs |
| J98.8 | Other specified respiratory disorders | Other RTIs |
| A36.3 | Cutaneous diphtheria | Skin infections |
| A46 | Erysipelas | Skin infections |
| K12.2 | Cellulitis and abscess of mouth | Skin infections |
| L00 | Staphylococcal scalded skin syndrome | Skin infections |
| L01 | Impetigo | Skin infections |
| L02 | Cutaneous abscess, furuncle and carbuncle | Skin infections |
| L03 | Cellulitis | Skin infections |
| L04 | Acute lymphadenitis of face, head and neck | Skin infections |
| L08.0 | Pyoderma (excl pyoderma gangrenosum) | Skin infections |
| L08.8 | Other spec local infections of skin and subcutaneous tissue | Skin infections |
| L08.9 | Local infection of skin and subcutaneous tissue unspecified | Skin infections |
| N30.0 | Cystitis | UTI-cystitis |
| N30.9 | Cystitis, unspecified | UTI-cystitis |
| N10 | Acute tubulo-interstitial nephritis | UTI-others |
| N12 | Tubulo-interstitial nephritis, not specified as acute or chronic | UTI-others |
| N13.6 | Pyenephrosis | UTI-others |
| N15.1 | Renal and perinephric abscess | UTI-others |
| N15.9 | Renal tubule-interstitial disease, unspecified | UTI-others |
| N16.0 | Renal tubule-interstitial disorders in infectious and parasitic diseases | UTI-others |
| N30.8 | Other cystitis - abscess of bladder | UTI-others |
| N39.0 | Urinary tract infection, site not specified | UTI-others |
| N41.0 | Acute prostatitis | UTI-others |
| N41.2 | Abscess of prostate | UTI-others |
| N41.3 | Prostatocystitis | UTI-others |
| N41.9 | Inflammatory disease of prostate, unspecified | UTI-others |

**Appendix 3.** List of Antimicrobials and Their Corresponding ATC Class, AWaRe Category, and WHO-EML Classification

| **Antimicrobials** | **Route of administration** | **Class** | **Derived AWaRe-Category** | **AWaRe Category-original** | **WHO-EML list** |
| --- | --- | --- | --- | --- | --- |
| Amikacin | Parenteral | Aminoglycosides | Access | Access | Yes |
| Gentamicin | Parenteral | Aminoglycosides | Access | Access | Yes |
| Chloramphenicol | Oral | Amphenicols | Access | Access | Yes |
| Chloramphenicol | Parenteral | Amphenicols | Access | Access | Yes |
| Enteromycin | Oral | Amphenicols | Access | Access | No |
| Thiamphenicol | Oral | Amphenicols | Access | Access | No |
| Thiamphenicol | Parenteral | Amphenicols | Access | Access | No |
| Amoxicillin/ Clavulanic acid | Oral | Beta-lactam/beta-lactamase-inhibitor | Access | Access | Yes |
| Amoxicillin/ Clavulanic acid | Parenteral | Beta-lactam/beta-lactamase-inhibitor | Access | Access | Yes |
| Ampicillin/ Sulbactam | Oral | Beta-lactam/beta-lactamase-inhibitor | Access | Access | No |
| Ampicillin/ Sulbactam | Parenteral | Beta-lactam/beta-lactamase-inhibitor | Access | Access | No |
| Cefadroxil | Oral | First-generation-cephalosporins | Access | Access | No |
| Cefalexin | Oral | First-generation-cephalosporins | Access | Access | Yes |
| Cefalexin | Parenteral | First-generation-cephalosporins | Access | Access | Yes |
| Cefapirin | Parenteral | First-generation-cephalosporins | Access | Access | No |
| Cefazolin | Parenteral | First-generation-cephalosporins | Access | Access | No |
| Cefradine | Oral | First-generation-cephalosporins | Access | Access | No |
| Cefradine | Parenteral | First-generation-cephalosporins | Access | Access | No |
| Metronidazole | Oral | Imidazoles | Access | Access | Yes |
| Metronidazole | Parenteral | Imidazoles | Access | Access | Yes |
| Nitazoxanide | Oral | Imidazoles | Access | Unclassified | No |
| Ornidazole | Oral | Imidazoles | Access | Access | No |
| Secnidazole | Oral | Imidazoles | Access | Access | No |
| Tinidazole | Oral | Imidazoles | Access | Access | No |
| Clindamycin | Oral | Lincosamides | Access | Access | Yes |
| Clindamycin | Parenteral | Lincosamides | Access | Access | Yes |
| Nifuroxazide | Oral | Nitrofuran-derivatives | Access | Unclassified | No |
| Nitrofurantoin | Oral | Nitrofuran-derivatives | Access | Access | Yes |
| Nitrofurazone | Parenteral | Nitrofuran-derivatives | Access | Unclassified | No |
| Amoxicillin | Oral | Penicillins | Access | Access | Yes |
| Amoxicillin | Parenteral | Penicillins | Access | Access | Yes |
| Ampicillin | Oral | Penicillins | Access | Access | Yes |
| Ampicillin | Parenteral | Penicillins | Access | Access | Yes |
| Bacampicillin | Oral | Penicillins | Access | Access | No |
| Benzathine-benzylpenicillin | Parenteral | Penicillins | Access | Access | Yes |
| Benzylpenicillin | Oral | Penicillins | Access | Access | Yes |
| Benzylpenicillin | Parenteral | Penicillins | Access | Access | Yes |
| Cloxacillin | Oral | Penicillins | Access | Access | No |
| Cloxacillin | Parenteral | Penicillins | Access | Access | No |
| Dicloxacillin | Oral | Penicillins | Access | Access | No |
| Flucloxacillin | Oral | Penicillins | Access | Access | No |
| Flucloxacillin | Parenteral | Penicillins | Access | Access | No |
| Oxacillin | Oral | Penicillins | Access | Access | No |
| Penicillin | Parenteral | Penicillins | Access | Access | Yes |
| Phenoxymethylpenicillin | Oral | Penicillins | Access | Access | Yes |
| Phenoxymethylpenicillin | Parenteral | Penicillins | Access | Access | No |
| Procaine-benzylpenicillin | Parenteral | Penicillins | Access | Access | No |
| Sulfadiazine | Oral | Sulfonamides | Access | Access | No |
| Sulfamethoxazole/ Trimethoprim | Oral | Sulfonamide-trimethoprim-combinations | Access | Access | Yes |
| Sulfamethoxazole/ Trimethoprim | Parenteral | Sulfonamide-trimethoprim-combinations | Access | Access | Yes |
| Doxycycline | Oral | Tetracyclines | Access | Access | No |
| Doxycycline | Parenteral | Tetracyclines | Access | Access | Yes |
| Tetracycline | Oral | Tetracyclines | Access | Access | No |
| Tetracycline | Parenteral | Tetracyclines | Access | Access | Yes |
| Amoxicillin/ Cloxacillin | Oral | Unclassified | Access | Not Recommended | No |
| Amoxicillin/ Dicloxacillin | Oral | Unclassified | Access | Not Recommended | No |
| Amoxicillin/ Flucloxacillin | Oral | Unclassified | Access | Not Recommended | No |
| Amoxicillin/ Flucloxacillin | Parenteral | Unclassified | Access | Not Recommended | No |
| Amoxicillin/ Metronidazole | Oral | Unclassified | Access | Not Recommended | No |
| Ampicillin/ Cloxacillin | Oral | Unclassified | Access | Not Recommended | No |
| Ampicillin/ Cloxacillin | Parenteral | Unclassified | Access | Not Recommended | No |
| Ampicillin/ Dicloxacillin | Oral | Unclassified | Access | Not Recommended | No |
| Ampicillin/ Dicloxacillin | Parenteral | Unclassified | Access | Not Recommended | No |
| Ampicillin/ Flucloxacillin | Oral | Unclassified | Access | Not Recommended | No |
| Benzylpenicillin/ Kanamycin | Parenteral | Unclassified | Access | Not Recommended | No |
| Benzylpenicillin/ Procaine | Parenteral | Unclassified | Access | Unclassified | No |
| Furazolidone/ Metronidazole | Oral | Unclassified | Access | Unclassified | No |
| Tigecycline | Parenteral | Glycylcyclines | Reserve | Reserve | No |
| Aztreonam | Oral | Monobactams | Reserve | Reserve | No |
| Linezolid | Oral | Oxazolidinones | Reserve | Reserve | Yes |
| Linezolid | Parenteral | Oxazolidinones | Reserve | Reserve | Yes |
| Tedizolid | Oral | Oxazolidinones | Reserve | Reserve | No |
| Fosfomycin | Parenteral | Phosphonics | Reserve | Reserve | Yes |
| Colistin | Oral | Polymyxins | Reserve | Reserve | No |
| Colistin | Parenteral | Polymyxins | Reserve | Reserve | Yes |
| Dibekacin | Parenteral | Aminoglycosides | Watch | Watch | No |
| Kanamycin | Parenteral | Aminoglycosides | Watch | Watch | No |
| Neomycin | Oral | Aminoglycosides | Watch | Watch | No |
| Tobramycin | Parenteral | Aminoglycosides | Watch | Watch | No |
| Piperacillin/ Tazobactam | Parenteral | Beta-lactam/beta-lactamase-inhibitor_anti-pseudomonal | Watch | Watch | No |
| Cilastatin/ Imipenem | Parenteral | Carbapenems | Watch | Watch | No |
| Doripenem | Parenteral | Carbapenems | Watch | Watch | No |
| Ertapenem | Parenteral | Carbapenems | Watch | Watch | No |
| Meropenem | Parenteral | Carbapenems | Watch | Watch | Yes |
| Ciprofloxacin | Oral | Fluoroquinolones | Watch | Watch | Yes |
| Ciprofloxacin | Parenteral | Fluoroquinolones | Watch | Watch | Yes |
| Enoxacin | Oral | Fluoroquinolones | Watch | Watch | No |
| Gatifloxacin | Oral | Fluoroquinolones | Watch | Watch | No |
| Gatifloxacin | Parenteral | Fluoroquinolones | Watch | Watch | No |
| Gemifloxacin | Oral | Fluoroquinolones | Watch | Watch | No |
| Levofloxacin | Oral | Fluoroquinolones | Watch | Watch | No |
| Levofloxacin | Parenteral | Fluoroquinolones | Watch | Watch | No |
| Lomefloxacin | Oral | Fluoroquinolones | Watch | Watch | No |
| Moxifloxacin | Oral | Fluoroquinolones | Watch | Watch | No |
| Moxifloxacin | Parenteral | Fluoroquinolones | Watch | Watch | No |
| Norfloxacin | Oral | Fluoroquinolones | Watch | Watch | No |
| Ofloxacin | Oral | Fluoroquinolones | Watch | Watch | No |
| Pefloxacin | Oral | Fluoroquinolones | Watch | Watch | No |
| Sparfloxacin | Oral | Fluoroquinolones | Watch | Watch | No |
| Cefepime | Parenteral | Fourth-generation-cephalosporins | Watch | Watch | No |
| Cefpirome | Parenteral | Fourth-generation-cephalosporins | Watch | Watch | No |
| Teicoplanin | Parenteral | Glycopeptides | Watch | Watch | No |
| Vancomycin | Parenteral | Glycopeptides | Watch | Watch | Yes |
| Lincomycin | Oral | Lincosamides | Watch | Watch | No |
| Lincomycin | Parenteral | Lincosamides | Watch | Watch | No |
| Azithromycin | Oral | Macrolides | Watch | Watch | Yes |
| Azithromycin | Parenteral | Macrolides | Watch | Watch | Yes |
| Clarithromycin | Oral | Macrolides | Watch | Watch | Yes |
| Clarithromycin | Parenteral | Macrolides | Watch | Watch | Yes |
| Erythromycin | Oral | Macrolides | Watch | Watch | No |
| Erythromycin | Parenteral | Macrolides | Watch | Watch | No |
| Roxithromycin | Oral | Macrolides | Watch | Watch | No |
| Roxithromycin | Parenteral | Macrolides | Watch | Watch | No |
| Spiramycin | Oral | Macrolides | Watch | Watch | No |
| Telithromycin | Oral | Macrolides | Watch | Watch | No |
| Nalidixic acid | Oral | Other quinolones | Watch | Unclassified | No |
| Piperacillin | Parenteral | Penicillins | Watch | Watch | No |
| Fosfomycin | Oral | Phosphonics | Watch | Watch | Yes |
| Pipemidic acid | Oral | Quinolones | Watch | Watch | No |
| Rifaximin | Oral | Rifamycins | Watch | Watch | No |
| Cefaclor | Oral | Second-generation-cephalosporins | Watch | Watch | No |
| Cefamandole | Oral | Second-generation-cephalosporins | Watch | Watch | No |
| Cefotiam | Oral | Second-generation-cephalosporins | Watch | Watch | No |
| Cefoxitin | Parenteral | Second-generation-cephalosporins | Watch | Watch | No |
| Cefprozil | Oral | Second-generation-cephalosporins | Watch | Watch | No |
| Cefuroxime | Oral | Second-generation-cephalosporins | Watch | Watch | Yes |
| Cefuroxime | Parenteral | Second-generation-cephalosporins | Watch | Watch | Yes |
| Fusidic acid | Oral | Steroid antibacterials | Watch | Watch | No |
| Minocycline | Oral | Tetracyclines | Watch | Watch | No |
| Oxytetracycline | Oral | Tetracyclines | Watch | Watch | No |
| Oxytetracycline | Parenteral | Tetracyclines | Watch | Watch | No |
| Cefdinir | Oral | Third-generation-cephalosporins | Watch | Watch | No |
| Cefditoren-pivoxil | Oral | Third-generation-cephalosporins | Watch | Watch | No |
| Cefixime | Oral | Third-generation-cephalosporins | Watch | Watch | Yes |
| Cefixime | Parenteral | Third-generation-cephalosporins | Watch | Watch | Yes |
| Cefoperazone | Parenteral | Third-generation-cephalosporins | Watch | Watch | No |
| Cefotaxime | Parenteral | Third-generation-cephalosporins | Watch | Watch | Yes |
| Cefpodoxime | Oral | Third-generation-cephalosporins | Watch | Watch | No |
| Ceftazidime | Parenteral | Third-generation-cephalosporins | Watch | Watch | Yes |
| Ceftizoxime | Parenteral | Third-generation-cephalosporins | Watch | Watch | No |
| Ceftriaxone | Oral | Third-generation-cephalosporins | Watch | Watch | Yes |
| Ceftriaxone | Parenteral | Third-generation-cephalosporins | Watch | Watch | Yes |
| Cefixime/ Clavulanic acid | Parenteral | Unclassified | Watch | Not Recommended | No |
| Cefoperazone/ Sulbactam | Parenteral | Unclassified | Watch | Not Recommended | No |
| Chloramphenicol/ Streptomycin | Oral | Unclassified | Watch | Unclassified | No |
| Ciprofloxacin/ Metronidazole | Oral | Unclassified | Watch | Not Recommended | No |
| Erythromycin/ Trimethoprim | Oral | Unclassified | Watch | Not Recommended | No |
| Metronidazole/ Spiramycin | Oral | Unclassified | Watch | Not Recommended | No |
| Metronidazole/ Spiramycin | Parenteral | Unclassified | Watch | Not Recommended | No |
| Norfloxacin/ Tinidazole | Oral | Unclassified | Watch | Unclassified | No |
| Phthalylsulfathiazole/ Streptomycin/ Diiodohydroxyquinoline/ Homatropine methylhydroxide | Oral | Unclassified | Watch | Unclassified | No |


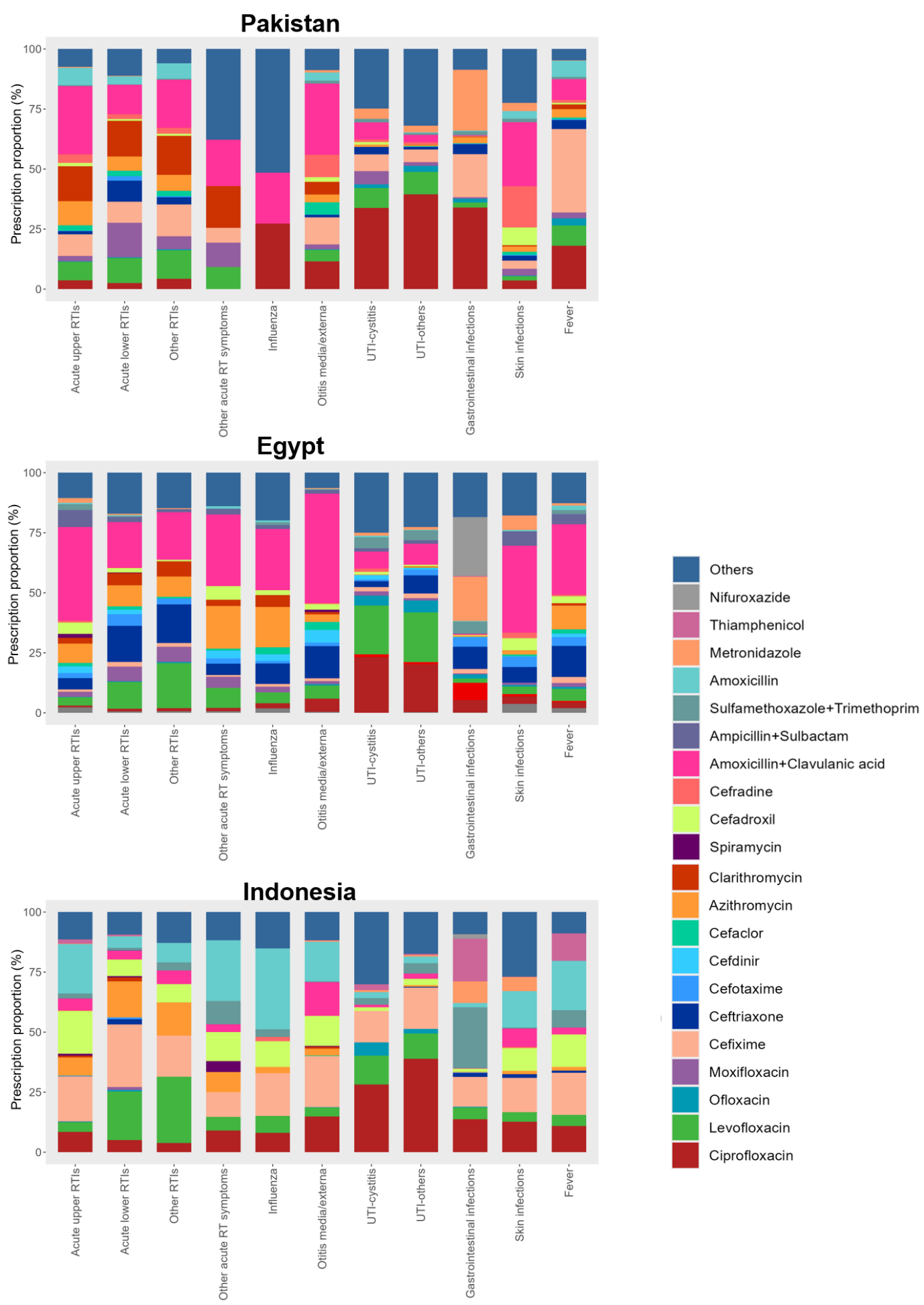


**Appendix 4.** Distribution of antibiotic prescribing by active substances and across countries. Source: Author analysis of IQVIA Medical Data Index (Medical Index of Pakistan (MIP), Egypt Medical Data Index (EMDI), Indonesia Medical Data Index (IMDI)) for the period 2017-2021, reflecting estimates of real world activity. Copyright IQVIA. All rights reserved.

**Appendix 5. WHO AWaRe antibiotic prescription proportional distribution, by prescriber’s speciality and by country.** Source: Author analysis of IQVIA Medical Data Index (Medical Index of Pakistan (MIP), Egypt Medical Data Index (EMDI), Indonesia Medical Data Index (IMDI)) for the period 2017-2021, reflecting estimates of real world activity. Copyright IQVIA. All rights reserved.

|  | **Antibiotic prescription proportion**  **N(%)** | **Distributional proportions of WHO-AWaRe antibiotics within the total antibiotic prescription** | | |
| --- | --- | --- | --- | --- |
|  |  | Accessantibiotics  N(%) | Watchantibiotics  N(%) | Reserve-antibiotics  N(%) |
| **Pakistan** |  |  |  |  |
| General Practitioners | 105321 (84.02) | 31098 (29.53) | 74083 (70.34) | 140 (0.13) |
| Paediatrics | 9808 (75.37) | 3327 (33.92) | 6472 (65.99) | 9 (0.09) |
| Internal Medicine | 8964 (86.96) | 1846 (20.59) | 7084 (79.03) | 34 (0.38) |
| ENT/ Otorhinolaryngology | 6660 (87.36) | 2278 (34.2) | 4364 (65.53) | 18 (0.27) |
| Pulmonology | 5766 (69.76) | 913 (15.83) | 4801 (83.26) | 52 (0.9) |
| Dermatology | 1105 (82.59) | 876 (79.28) | 193 (17.47) | 36 (3.26) |
| Surgery | 2673 (90.61) | 860 (32.17) | 1721 (64.38) | 92 (3.44) |
| Resident Medical Officers | 19841 (79.4) | 6729 (33.91) | 12992 (65.48) | 120 (0.6) |
| Others | 7819 (71.22) | 1546 (19.77) | 6242 (79.83) | 31 (0.4) |
| **Egypt** |  |  |  |  |
| General Practitioners | 12347 (83.34) | 5909 (47.86) | 6383 (51.7) | 55 (0.45) |
| Paediatrics | 14777 (71.74) | 8128 (55) | 6536 (44.23) | 113 (0.76) |
| Internal Medicine | 8575 (79.96) | 3192 (37.22) | 5308 (61.9) | 75 (0.87) |
| ENT/ Otorhinolaryngology | 13217 (87.17) | 7450 (56.37) | 5735 (43.39) | 32 (0.24) |
| Pulmonology | 8887 (84.03) | 2292 (25.79) | 6223 (70.02) | 372 (4.19) |
| Dermatology | 1279 (86.83) | 912 (71.31) | 329 (25.72) | 38 (2.97) |
| Surgery | 2707 (91.08) | 1532 (56.59) | 1064 (39.31) | 111 (4.1) |
| Nephrology/Urology | 4414 (91.39) | 850 (19.26) | 3556 (80.56) | 8 (0.18) |
| Others | 4638 (75.59) | 2275 (49.05) | 2307 (49.74) | 56 (1.21) |
| **Indonesia** |  |  |  |  |
| General Practitioners | 35887 (70.44) | 19110 (53.25) | 16752 (46.68) | 25 (0.07) |
| Paediatrics | 7981 (53.33) | 3997 (50.08) | 3940 (49.37) | 44 (0.55) |
| Internal Medicine | 4419 (77.68) | 1015 (22.97) | 3401 (76.96) | 3 (0.07) |
| ENT/ Otorhinolaryngology | 7444 (73.1) | 3167 (42.54) | 4276 (57.44) | 1 (0.01) |
| Pulmonology | 3017 (74.86) | 460 (15.25) | 2542 (84.26) | 15 (0.5) |
| Dermatology | 1114 (81.85) | 570 (51.17) | 544 (48.83) | 0 (0) |
| Surgery | 1640 (90.71) | 438 (26.71) | 1201 (73.23) | 1 (0.06) |
| Others | 2608 (68.22) | 920 (35.28) | 1682 (64.49) | 6 (0.23) |

**Appendix 6.** Variability in Watch antibiotic prescription proportion among prescribers within the same speciality by country. Source: Author analysis of IQVIA Medical Data Index (Medical Index of Pakistan (MIP), Egypt Medical Data Index (EMDI), Indonesia Medical Data Index (IMDI)) for the period 2017-2021, reflecting estimates of real world activity. Copyright IQVIA. All rights reserved.

**PAKISTAN**

| **Specialisation** | **Mean (standard deviation)** | **Coefficient of variation** | **Median (25^th^-75^th^ Percentile)** | **Interquartile range** |
| --- | --- | --- | --- | --- |
| General Practice | 70 (12.4) | 0.18 | 70.3 (63.8-77.6) | 13.8 |
| Paediatrics | 64.3 (18.8) | 0.29 | 63.2 (53.8-74.7) | 20.9 |
| Internal Medicine | 79.1 (10.1) | 0.13 | 79.4 (72.2-83.3) | 11.1 |
| ENT/Otorhinolaryngology | 66 (14.7) | 0.22 | 67.6 (53.6-74.8) | 21.2 |
| Pulmonology | 82.9 (10.7) | 0.13 | 84.1 (74.3-89.8) | 15.5 |
| Dermatology | 23 (17.1) | 0.74 | 21.9 (10-31.8) | 21.9 |
| Surgery | 62.4 (16) | 0.26 | 66.3 (49.1-73.3) | 24.2 |
| Resident Medicine | 68.2 (12.4) | 0.18 | 69.4 (57.7-76) | 18.3 |
| Others | 75.4 (16.7) | 0.22 | 78.8 (67.3-86) | 18.7 |

**EGYPT**

| **Specialisation** | **Mean (standard deviation)** | **Coefficient of variation** | **Median (25^th^-75^th^ Percentile)** | **Interquartile range** |
| --- | --- | --- | --- | --- |
| General Practitioners | 51.1 (20.2) | 0.40 | 50 (38-66.3) | 28.2 |
| Paediatrics | 42.3 (17.1) | 0.40 | 40.1 (27.8-53.9) | 26.1 |
| Internal Medicine | 63.6 (16.4) | 0.26 | 66 (52.8-75.5) | 22.7 |
| ENT/Otorhinolaryngology | 44.5 (18.4) | 0.41 | 43.9 (34.2-55) | 20.8 |
| Pulmonology | 74.8 (14.9) | 0.20 | 76.3 (63-87) | 24 |
| Dermatology | 33.3 (23.5) | 0.71 | 28.6 (14.3-43.5) | 29.2 |
| Surgery | 39.5 (19.2) | 0.49 | 40.8 (23.7-55.5) | 31.7 |
| Nephrology/Urology | 79.5 (14.3) | 0.18 | 80.6 (73.5-89.9) | 16.4 |
| Others | 52.8 (23.7) | 0.45 | 52.9 (33.3-73.7) | 40.4 |

**INDONESIA**

| **Specialisation** | **Mean (standard deviation)** | **Coefficient of variation** | **Median (25^th^-75^th^ Percentile)** | **Interquartile range** |
| --- | --- | --- | --- | --- |
| General Practitioners | 47.1 (23.3) | 0.50 | 45.8 (29.7-63.2) | 33.6 |
| Paediatrics | 52.7 (25.2) | 0.48 | 55.2 (32.1-72.1) | 39.9 |
| Internal Medicine | 75.6 (18.2) | 0.24 | 80 (65.3-89.4) | 24.1 |
| ENT/Otorhinolaryngology | 56.1 (24.4) | 0.43 | 56.3 (34.2-76.5) | 42.4 |
| Pulmonology | 83 (14.5) | 0.18 | 88.9 (74.6-95) | 20.4 |
| Dermatology | 55.3 (26.6) | 0.48 | 60 (35.4-75) | 39.6 |
| Surgery | 68.9 (21) | 0.31 | 73.9 (57-85.5) | 28.5 |
| Others | 64.5 (26.9) | 0.42 | 73 (41.3-87.5) | 46.2 |

**Appendix 7.** The number and proportion of infectious diagnoses, along with their corresponding categorization indicating the need for antibiotic treatment to manage the disease. Source: Author analysis of IQVIA Medical Data Index (Medical Index of Pakistan (MIP), Egypt Medical Data Index (EMDI), Indonesia Medical Data Index (IMDI)) for the period 2017-2021, reflecting estimates of real world activity. Copyright IQVIA. All rights reserved.

A: always requiring antibiotic; S: sometimes requiring antibiotics; N: never requiring antibiotics.
Source: Chua K-P, Fischer MA, Linder JA. Appropriateness of outpatient antibiotic prescribing among privately insured US patients: ICD-10-CM based cross sectional study. *BMJ* 2019; **364**: k5092.

**PAKISTAN**

| **Infection group** | **Diagnosis** | **category** | **N (%)** |
| --- | --- | --- | --- |
| Acute upper RTIs | Acute pharyngitis | S | 17 237 (8.41) |
| Acute upper RTIs | Acute upper respiratory infections of multiple and unspecified sites | N | 15 022 (7.33) |
| Acute upper RTIs | Acute tonsillitis | S | 14 817 (7.23) |
| Acute upper RTIs | Acute sinusitis | S | 3 531 (1.72) |
| Acute upper RTIs | Acute nasopharyngitis | N | 2 565 (1.25) |
| Acute upper RTIs | Acute laryngitis and tracheitis | S | 1 230 (0.6) |
| Acute upper RTIs | Peritonsillar abscess | A | 89 (0.04) |
| Acute upper RTIs | Acute obstructive laryngitis [croup] and epiglottitis | S | 32 (0.02) |
| Acute lower RTIs | Acute bronchitis | N | 6 268 (3.06) |
| Acute lower RTIs | Pneumonia, organism unspecified | A | 4 255 (2.08) |
| Acute lower RTIs | Unspecified acute lower respiratory infection | S | 2 204 (1.08) |
| Acute lower RTIs | COPD with acute exacerbation, unspecified | S | 685 (0.33) |
| Acute lower RTIs | Pneumonia due to Streptococcus pneumoniae | A | 237 (0.12) |
| Acute lower RTIs | Acute bronchiolitis | N | 181 (0.09) |
| Acute lower RTIs | Bronchitis, not specified as acute or chronic | N | 139 (0.07) |
| Acute lower RTIs | Bacterial pneumonia, not elsewhere classified | A | 33 (0.02) |
| Influenza | Influenza, virus not identified | S | 13 691 (6.68) |
| Other RTIs | Other specified respiratory disorders | N | 28 600 (13.96) |
| Other respiratory acute symptoms | Cough | N | 43 389 (21.18) |
| Other respiratory acute symptoms | Chest pain, unspecified | N | 2 834 (1.38) |
| Other respiratory acute symptoms | Pain in throat | N | 832 (0.41) |
| Other respiratory acute symptoms | Abnormal sputum | N | 68 (0.03) |
| Other respiratory acute symptoms | Chest pain on breathing | N | 12 (0.01) |
| Mastoiditis and other ear infection complications | Otitis media, unspecified | S | 3 130 (1.53) |
| Mastoiditis and other ear infection complications | Acute suppurative otitis media | S | 1 739 (0.85) |
| Mastoiditis and other ear infection complications | Other infective otitis externa | S | 1 050 (0.51) |
| Mastoiditis and other ear infection complications | Abscess of external ear | S | 573 (0.28) |
| Mastoiditis and other ear infection complications | Nonsuppurative otitis media, unspecified | N | 115 (0.06) |
| Mastoiditis and other ear infection complications | Mastoiditis and related conditions | S | 103 (0.05) |
| Gastrointestinal infections | Other gastroenteritis and colitis of infectious and unspecified origin | S | 32 545 (15.88) |
| Gastrointestinal infections | Typhoid and paratyphoid fevers | A | 12 035 (5.87) |
| Gastrointestinal infections | Amoebiasis | A | 918 (0.45) |
| Gastrointestinal infections | Shigellosis | S | 183 (0.09) |
| Gastrointestinal infections | Other protozoal intestinal diseases | S | 113 (0.06) |
| Gastrointestinal infections | Other bacterial intestinal infections | S | 44 (0.02) |
| Gastrointestinal infections | Cholera | S | 28 (0.01) |
| Gastrointestinal infections | Viral and other specified intestinal infections | S | 11 (0.01) |
| UTI-cystitis | Cystitis, unspecified | S | 766 (0.37) |
| UTI-cystitis | Cystitis | A | 222 (0.11) |
| UTI-others | Urinary tract infection, site not specified | A | 20 572 (10.04) |
| UTI-others | Acute tubulo-interstitial nephritis | A | 129 (0.06) |
| UTI-others | Inflammatory disease of prostate, unspecified | S | 25 (0.01) |
| UTI-others | Other cystitis - abscess of bladder | S | 7 (0) |
| Skin infections | Cutaneous abscess, furuncle and carbuncle | S | 3 077 (1.5) |
| Skin infections | Cellulitis | S | 1 619 (0.79) |
| Skin infections | Impetigo | S | 851 (0.42) |
| Skin infections | Local infection of skin and subcutaneous tissue unspecified | S | 770 (0.38) |
| Skin infections | Other spec local infections of skin and subcutaneous tissue | S | 374 (0.18) |
| Skin infections | Acute lymphadenitis | S | 208 (0.1) |
| Skin infections | Pyoderma (excl pyoderma gangrenosum) | S | 103 (0.05) |
| Skin infections | Erysipelas | A | 34 (0.02) |
| Skin infections | Cellulitis and abscess of mouth | A | 11 (0.01) |
| Fever | Fever of other and unknown origin | N | 59 936 (29.25) |
|  | Others |  | 14 (0.01) |

**EGYPT**

| **Infection group** | **Diagnosis** | **category** | **N (%)** |
| --- | --- | --- | --- |
| Acute upper RTIs | Acute tonsillitis | S | 9 284 (10.64) |
| Acute upper RTIs | Acute pharyngitis | S | 6 244 (7.15) |
| Acute upper RTIs | Acute nasopharyngitis | N | 5 369 (6.15) |
| Acute upper RTIs | Acute upper respiratory infections of multiple and unspecified sites | N | 2 826 (3.24) |
| Acute upper RTIs | Acute sinusitis | S | 2 356 (2.7) |
| Acute upper RTIs | Acute laryngitis and tracheitis | S | 995 (1.14) |
| Acute upper RTIs | Peritonsillar abscess | A | 143 (0.16) |
| Acute upper RTIs | Acute obstructive laryngitis [croup] and epiglottitis | S | 131 (0.15) |
| Acute lower RTIs | Acute bronchitis | N | 10 099 (11.57) |
| Acute lower RTIs | Bronchitis, not specified as acute or chronic | N | 2 925 (3.35) |
| Acute lower RTIs | Pneumonia, organism unspecified | A | 1 926 (2.21) |
| Acute lower RTIs | Acute bronchiolitis | N | 1 039 (1.19) |
| Acute lower RTIs | COPD with acute exacerbation, unspecified | S | 669 (0.77) |
| Acute lower RTIs | Unspecified acute lower respiratory infection | S | 134 (0.15) |
| Acute lower RTIs | Viral pneumonia, not elsewhere classified | N | 93 (0.11) |
| Acute lower RTIs | COPD with acute lower respiratory tract infection | S | 38 (0.04) |
| Acute lower RTIs | Bacterial pneumonia, not elsewhere classified | A | 17 (0.02) |
| Influenza | Influenza, virus not identified | S | 1 463 (1.68) |
| Other RTIs | Other specified respiratory disorders | N | 1 357 (1.55) |
| Other RTIs | Respiratory infections, not elsewhere classified (Respiratory (tract) infections not specified as acute, chronic, lower, or upper) | S | 13 (0.01) |
| Other respiratory acute symptoms | Cough | N | 4 010 (4.59) |
| Other respiratory acute symptoms | Chest pain, unspecified | N | 1 097 (1.26) |
| Other respiratory acute symptoms | Chest pain on breathing | N | 205 (0.23) |
| Other respiratory acute symptoms | Abnormal sputum | N | 129 (0.15) |
| Mastoiditis and other ear infection complications | Otitis media, unspecified | S | 4 611 (5.28) |
| Mastoiditis and other ear infection complications | Otitis externa, unspecified | S | 1 382 (1.58) |
| Mastoiditis and other ear infection complications | Nonsuppurative otitis media, unspecified | N | 608 (0.7) |
| Mastoiditis and other ear infection complications | Acute suppurative otitis media | S | 175 (0.2) |
| Mastoiditis and other ear infection complications | Suppurative otitis media, unspecified | S | 153 (0.18) |
| Mastoiditis and other ear infection complications | Abscess of external ear | S | 137 (0.16) |
| Mastoiditis and other ear infection complications | Other acute nonsuppurative otitis media | N | 92 (0.11) |
| Mastoiditis and other ear infection complications | Malignant otitis externa | A | 74 (0.08) |
| Mastoiditis and other ear infection complications | Acute serous otitis media | N | 63 (0.07) |
| Mastoiditis and other ear infection complications | Other infective otitis externa | S | 41 (0.05) |
| Mastoiditis and other ear infection complications | Mastoiditis and related conditions | S | 24 (0.03) |
| Mastoiditis and other ear infection complications | Other otitis externa | S | 23 (0.03) |
| Mastoiditis and other ear infection complications | Other disorders of middle ear mastoid | N | 14 (0.02) |
| Gastrointestinal infections | Other gastroenteritis and colitis of infectious and unspecified origin | S | 11 426 (13.09) |
| Gastrointestinal infections | Amoebiasis | A | 835 (0.96) |
| Gastrointestinal infections | Typhoid and paratyphoid fevers | A | 814 (0.93) |
| Gastrointestinal infections | Other protozoal intestinal diseases | S | 181 (0.21) |
| Gastrointestinal infections | Other bacterial intestinal infections | S | 70 (0.08) |
| Gastrointestinal infections | Viral and other specified intestinal infections | S | 46 (0.05) |
| Gastrointestinal infections | Shigellosis | S | 43 (0.05) |
| Gastrointestinal infections | Other bacterial foodborne intoxications, not elsewhere classified | S | 10 (0.01) |
| UTI-cystitis | Cystitis, unspecified | S | 1 513 (1.73) |
| UTI-cystitis | Cystitis | A | 796 (0.91) |
| UTI-others | Urinary tract infection, site not specified | A | 4 831 (5.53) |
| UTI-others | Orchitis and epidymitis | S | 972 (1.11) |
| UTI-others | Inflammatory disease of prostate, unspecified | S | 511 (0.59) |
| UTI-others | Tubulo-interstitial nephritis, not specified as acute or chronic | N | 262 (0.3) |
| UTI-others | Acute prostatitis | A | 227 (0.26) |
| UTI-others | Acute tubulo-interstitial nephritis | A | 117 (0.13) |
| UTI-others | Prostatocystitis | S | 50 (0.06) |
| UTI-others | Salpingitis and oophoritis, unspecified | A | 33 (0.04) |
| UTI-others | Renal tubule-interstitial disease, unspecified | N | 20 (0.02) |
| UTI-others | Other cystitis - abscess of bladder | S | 15 (0.02) |
| UTI-others | Renal and perinephric abscess | A | 14 (0.02) |
| UTI-others | Acute salpingitis and oophoritis | A | 6 (0.01) |
| UTI-others | Pyenephrosis | N | 6 (0.01) |
| Skin infections | Cutaneous abscess, furuncle and carbuncle | S | 2 318 (2.66) |
| Skin infections | Cellulitis | S | 1 652 (1.89) |
| Skin infections | Impetigo | S | 602 (0.69) |
| Skin infections | Local infection of skin and subcutaneous tissue unspecified | S | 318 (0.36) |
| Skin infections | Erysipelas | A | 289 (0.33) |
| Skin infections | Acute lymphadenitis | S | 130 (0.15) |
| Skin infections | Pyoderma (excl pyoderma gangrenosum) | S | 99 (0.11) |
| Skin infections | Staphylococcal scalded skin syndrome | A | 86 (0.1) |
| Skin infections | Cellulitis and abscess of mouth | A | 69 (0.08) |
| Skin infections | Other spec local infections of skin and subcutaneous tissue | S | 39 (0.04) |
| Fever | Fever of other and unknown origin | N | 7 009 (8.03) |
|  | Other | | 22 (0.03) |

**INDONESIA**

| **Infection group** | **Diagnosis** | **Category** | **N (%)** |
| --- | --- | --- | --- |
| Acute upper RTIs | Acute upper respiratory infections of multiple and unspecified sites | N | 26 320 (28.36) |
| Acute upper RTIs | Acute nasopharyngitis | N | 10 468 (11.28) |
| Acute upper RTIs | Acute pharyngitis | S | 8 075 (8.7) |
| Acute upper RTIs | Acute tonsillitis | S | 1 607 (1.73) |
| Acute upper RTIs | Acute laryngitis and tracheitis | S | 441 (0.48) |
| Acute upper RTIs | Peritonsillar abscess | A | 118 (0.13) |
| Acute upper RTIs | Acute sinusitis | S | 112 (0.12) |
| Acute upper RTIs | Acute obstructive laryngitis [croup] and epiglottitis | S | 25 (0.03) |
| Acute upper RTIs | Retropharyngeal and parapharyngeal abscess | A | 10 (0.01) |
| Acute lower RTIs | Pneumonia, organism unspecified | A | 3 118 (3.36) |
| Acute lower RTIs | Bronchitis, not specified as acute or chronic | N | 2 355 (2.54) |
| Acute lower RTIs | Acute bronchitis | N | 826 (0.89) |
| Acute lower RTIs | COPD with acute exacerbation, unspecified | S | 216 (0.23) |
| Acute lower RTIs | Unspecified acute lower respiratory infection | S | 132 (0.14) |
| Acute lower RTIs | Acute bronchiolitis | N | 103 (0.11) |
| Acute lower RTIs | Bacterial pneumonia, not elsewhere classified | A | 27 (0.03) |
| Influenza | Influenza, virus not identified | S | 1 063 (1.15) |
| Other RTIs | Other specified respiratory disorders | N | 464 (0.5) |
| Other respiratory acute symptoms | Cough | N | 1 026 (1.11) |
| Other respiratory acute symptoms | Chest pain, unspecified | N | 325 (0.35) |
| Other respiratory acute symptoms | Abnormal sputum | N | 12 (0.01) |
| Mastoiditis and other ear infection complications | Otitis media, unspecified | S | 2 698 (2.91) |
| Mastoiditis and other ear infection complications | Otitis externa, unspecified | S | 2 617 (2.82) |
| Mastoiditis and other ear infection complications | Nonsuppurative otitis media, unspecified | N | 538 (0.58) |
| Mastoiditis and other ear infection complications | Other infective otitis externa | S | 342 (0.37) |
| Mastoiditis and other ear infection complications | Suppurative otitis media, unspecified | S | 138 (0.15) |
| Mastoiditis and other ear infection complications | Acute suppurative otitis media | S | 119 (0.13) |
| Mastoiditis and other ear infection complications | Abscess of external ear | S | 80 (0.09) |
| Mastoiditis and other ear infection complications | Mastoiditis and related conditions | S | 49 (0.05) |
| Mastoiditis and other ear infection complications | Other acute nonsuppurative otitis media | N | 45 (0.05) |
| Mastoiditis and other ear infection complications | Other otitis externa | S | 12 (0.01) |
| Gastrointestinal infections | Other gastroenteritis and colitis of infectious and unspecified origin | S | 11 730 (12.64) |
| Gastrointestinal infections | Typhoid and paratyphoid fevers | A | 3 486 (3.76) |
| Gastrointestinal infections | Amoebiasis | A | 246 (0.27) |
| Gastrointestinal infections | Shigellosis | S | 62 (0.07) |
| Gastrointestinal infections | Viral and other specified intestinal infections | S | 19 (0.02) |
| Gastrointestinal infections | Other bacterial foodborne intoxications, not elsewhere classified | S | 17 (0.02) |
| Gastrointestinal infections | Other bacterial intestinal infections | S | 17 (0.02) |
| Gastrointestinal infections | Cholera | S | 7 (0.01) |
| UTI-cystitis | Cystitis, unspecified | S | 643 (0.69) |
| UTI-cystitis | Cystitis | A | 87 (0.09) |
| UTI-others | Urinary tract infection, site not specified | A | 4 853 (5.23) |
| UTI-others | Orchitis and epidymitis | S | 215 (0.23) |
| UTI-others | Salpingitis and oophoritis, unspecified | A | 176 (0.19) |
| UTI-others | Tubulo-interstitial nephritis, not specified as acute or chronic | N | 30 (0.03) |
| UTI-others | Acute tubulo-interstitial nephritis | A | 18 (0.02) |
| UTI-others | Inflammatory disease of prostate, unspecified | S | 15 (0.02) |
| Skin infections | Cutaneous abscess, furuncle and carbuncle | S | 2 596 (2.8) |
| Skin infections | Cellulitis | S | 1 291 (1.39) |
| Skin infections | Pyoderma (excl pyoderma gangrenosum) | S | 792 (0.85) |
| Skin infections | Impetigo | S | 666 (0.72) |
| Skin infections | Staphylococcal scalded skin syndrome | A | 280 (0.3) |
| Skin infections | Local infection of skin and subcutaneous tissue unspecified | S | 74 (0.08) |
| Skin infections | Acute lymphadenitis | S | 72 (0.08) |
| Skin infections | Erysipelas | A | 48 (0.05) |
| Skin infections | Cellulitis and abscess of mouth | A | 39 (0.04) |
| Fever | Fever of other and unknown origin | N | 3 966 (4.27) |
|  | Other |  | 39 (0.04) |

**Appendix 8.** The number of antimicrobial agents, antimicrobial products, and suppliers identified in the prescriptions submitted by participating doctors, categorised by WHO-AWaRe classification from each country included in the study. Source: Author analysis of IQVIA Medical Data Index (Medical Index of Pakistan (MIP), Egypt Medical Data Index (EMDI), Indonesia Medical Data Index (IMDI)) for the period 2017-2021, reflecting estimates of real world activity. Copyright IQVIA. All rights reserved.

|  | **Pakistan** | **Egypt** | **Indonesia** |
| --- | --- | --- | --- |
| **Number of antimicrobial agents ^a^**  Access  Watch  Reserve | 23  42  2 | 35  46  3 | 23  36  4 |
| **Number of antimicrobial products ^b^**  Access  Watch  Reserve | 238  1037  18 | 295  407  20 | 423  363  6 |
| **Number of antimicrobial suppliers**  Access  Watch  Reserve | 95  255  13 | 92  167  19 | 79  71  4 |

Antimicrobial agents are the chemical substances of antibiotics, and the antimicrobial products are the marketed items

**Appendix 9.** Factors associated with antibiotic prescribing decisions for patients in Indonesia, with settings classified into doctor’s clinic, governmental hospitals and private hospitals. Source: Author analysis of IQVIA Medical Data Index (Indonesia Medical Data Index (IMDI))), reflecting estimates of real world activity. Copyright IQVIA. All rights reserved.


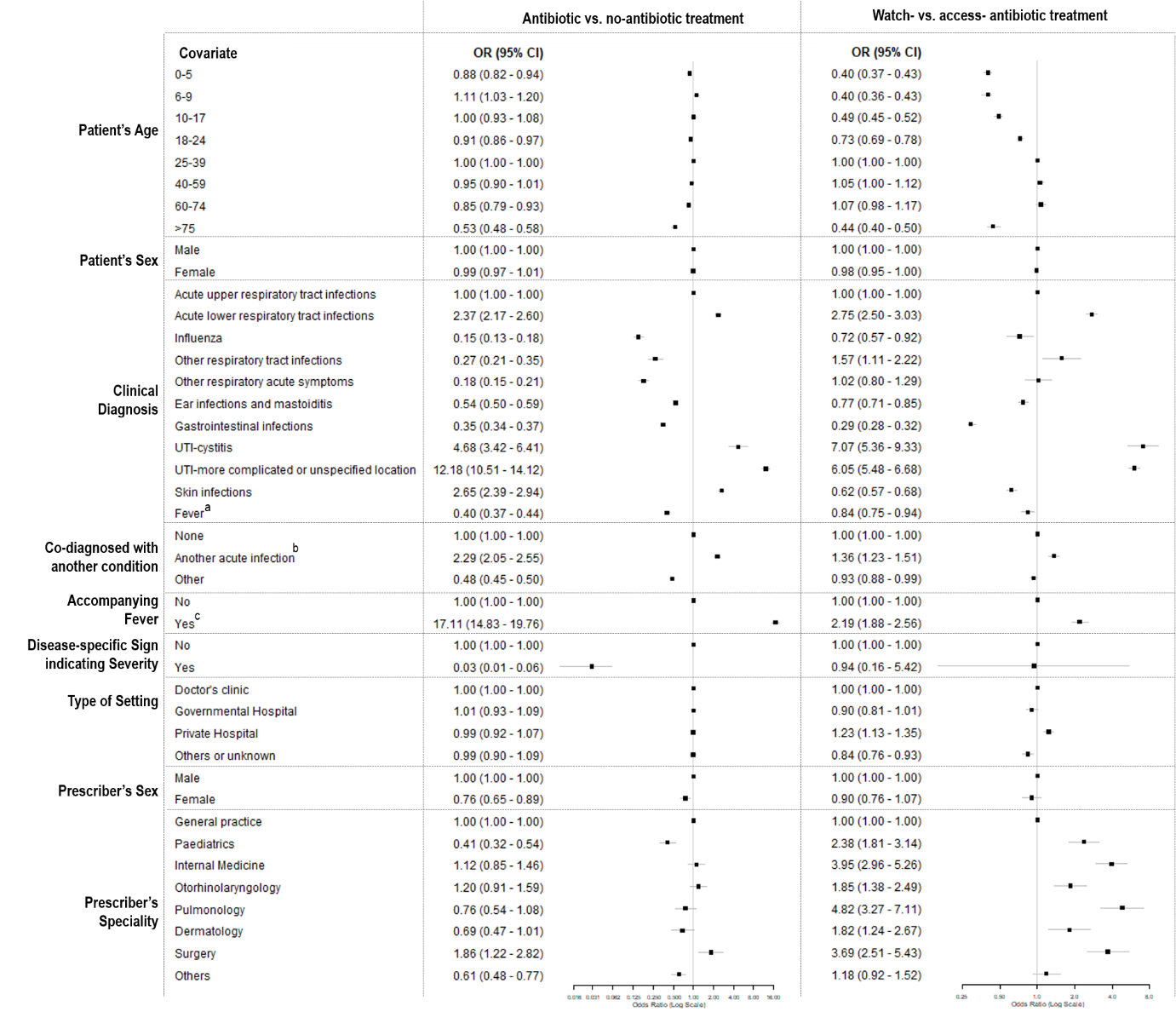


a: cases with single diagnosis of fever. b: cases with more than one diagnosis of acute infections. c: cases with

fever accompanying an acute infection.
